# Thiazole Functionalization of Thiosemicarbazone for Cu(II) Complexation: Moving toward Highly Efficient Anticancer Drugs with Promising Oral Bioavailability

**DOI:** 10.3390/molecules29163832

**Published:** 2024-08-13

**Authors:** Song-Yu Luo, Chun-Mei Zeng, Ping Xu, Ye Ning, Meng-Lin Dong, Wen-Hua Zhang, Guangliang Yu

**Affiliations:** 1 College of Chemistry, Chemical Engineering, and Materials Science, Soochow University, Suzhou 215123, China; songyuluo550@163.com (S.-Y.L.); 20214209084@stu.suda.edu.cn (C.-M.Z.); 15854833290@163.com (Y.N.); 20234209141@stu.suda.edu.cn (M.-L.D.); 2 Suzhou Degen Bio-Medical Co., Ltd., No. 1 Huayun Road, Suzhou Industrial Park, Suzhou 215000, China; 13262880543@163.com

**Keywords:** thiosemicarbazone, anticancer drug, copper complex, reactive oxygen species, pharmacokinetics

## Abstract

In this work, we report the synthesis of a new thiosemicarbazone-based drug of N′-(di(pyridin-2-yl)methylene)-4-(thiazol-2-yl)piperazine-1-carbothiohydrazide (H**L**) featuring a thiazole spectator for efficient coordination with Cu(II) to give [CuCl(**L**)]_2_ (**1**) and [Cu(NO_3_)(**L**)]_2_ (**2**). Both **1** and **2** exhibit dimeric structures ascribed to the presence of di-2-pyridylketone moieties that demonstrate dual functions of chelation and intermolecular bridging. H**L**, **1**, and **2** are highly toxic against hepatocellular carcinoma cell lines Hep-G2, PLC/PRF/5, and HuH-7 with half maximal inhibitory concentration (IC_50_) values as low as 3.26 nmol/mL (H**L**), 2.18 nmol/mL (**1**), and 2.54 × 10^−5^ nmol/mL (**2**) for PLC/PRF/5. While the free ligand H**L** may elicit its anticancer effect via the sequestration of bio-relevant metal ions (i.e., Fe^3+^ and Cu^2+^), **1** and **2** are also capable of generating cytotoxic reactive oxygen species (ROS) to inhibit cancer cell proliferation. Our preliminary pharmacokinetic studies revealed that oral administration (per os, PO) of H**L** has a significantly longer half-life *t*_1/2_ of 21.61 ± 9.4 h, nearly doubled as compared with that of the intravenous (i.v.) administration of 11.88 ± 1.66 h, certifying H**L** as an effective chemotherapeutic drug via PO administration.

## 1. Introduction

Thiosemicarbazones (TSCs) are a class of Schiff base derivatives that exhibit diverse biologically beneficial activities, such as antibacterial, antiviral, and enzyme inhibitory activities [1,2]. Most notably, TSCs have long been considered as a potential class of anticancer drug candidates for a broad spectrum of cancers through a wide range of action mechanisms, such as ribonuclease reductase and topoisomerase II inhibition [3,4,5,6]. Other mechanisms include the quick sequestration of cell-proliferative-dependent ions (i.e., Fe^3+^ and Cu^2+^) to elicit the anticancer effect [4]. The anticancer effect of TSCs is primarily linked to, and profoundly affected by, their structures that feature an S, N chelator [4,5]. In this regard, the additional introduction of an N coordination site, such as α-pyridyl, serves as an advantage, as it forms a more stable N, N, S pincer-like chelation that results in more effective ion removal [7]. In this sense, TSCs are particularly effective for treating iron overload diseases such as leukemia and neuroblastoma [8]. Recently, findings have also suggested that, upon the capturing of metal ions by TSCs, a different anticancer mechanism based on reactive oxygen species (ROS) chemistry can be commenced by taking advantage of the unique characteristics of the tumor microenvironment (TME), such as the overexpression of H_2_O_2_ and GSH [9,10,11,12]. The ROS generation can subsequently strengthen the chemotherapeutic outcome of TSCs.

The promising potential of TSCs as anticancer drugs has been manifested by several drugs that successfully entered clinical trials, such as 3-aminopyridine-2-carboxaldehyde thiosemicarbazone (Triapine), 5-hydroxypicolinaldehyde thiosemicarbazone (5-HP), and 4-(pyridine-2-yl)-N-([(8E)-5,6,7,8-tetrahydroquinolin-8-ylidene]amino)piperazine-1-carbothioamide (COTI-2) (Chart 1). Nevertheless, numerous side effects were also associated with these drugs, i.e., the formation of methemoglobin hypoxia by Triapine, [13] severe hematological and gastrointestinal side effects for 5-HP, [14] and drug resistance and other adverse effects (i.e., nausea, vomiting, and fatigue) for COTI-2 [15,16].

**Scheme 1:**
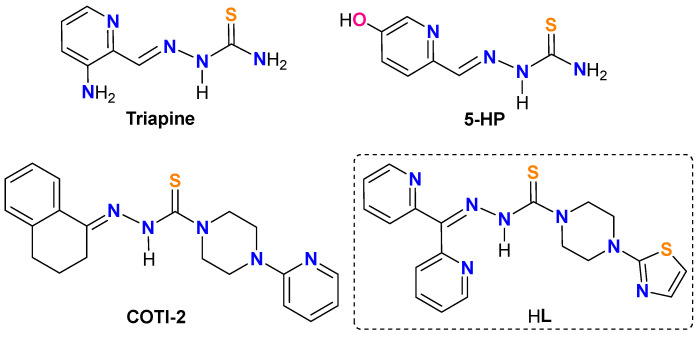
Molecular diagrams of Triapine, 5-HP, COTI-2, and H**L**.

In this work, we report the synthesis of a novel TSC-based anticancer drug of N′-(di(pyridin-2-yl)methylene)-4-(thiazol-2-yl)piperazine-1-carbothiohydrazide (H**L**) featuring di-2-pyridylketone for chelating–bridging and a thiazole spectator (Scheme 1). Ligand H**L** can readily associate Cu^2+^ to give the corresponding coordination complexes of [CuCl(**L**)]_2_ (**1**), and [Cu(NO_3_)(**L**)]_2_ (**2**). The structures of H**L**, **1**, and **2** were confirmed by various spectroscopic techniques and ultimately authenticated by single-crystal X-ray diffraction studies. H**L**, **1**, and **2** are highly toxic against hepatocellular carcinoma cell lines Hep-G2, PLC/PRF/5, and HuH-7. Notably, these species are extremely toxic for PLC/PRF/5, with the corresponding half maximal inhibitory concentration (IC_50_) values being as low as 3.26 nmol/mL (H**L**), 2.18 nmol/mL (1), and 2.54 × 10^−5^ nmol/mL (2). Our preliminary pharmacokinetic studies of H**L** further revealed that oral administration (per os, PO) exhibits a significantly longer half-life *t*_1/2_ of 21.61 ± 9.4 h as compared with that of the intravenous (i.v.) administration of 11.88 ± 1.66 h. These results highlight that H**L** and its relevant functional coordination complexes can be promising chemotherapeutic drugs for oral administration.

## 2. Results and Discussion

### 2.1. Synthesis and X-ray Structure Characterization of H**L**, [CuCl(**L**)]_2_ (***1***), and [Cu(NO_3_)(**L**)]_2_ (***2***)

**Scheme 2:**
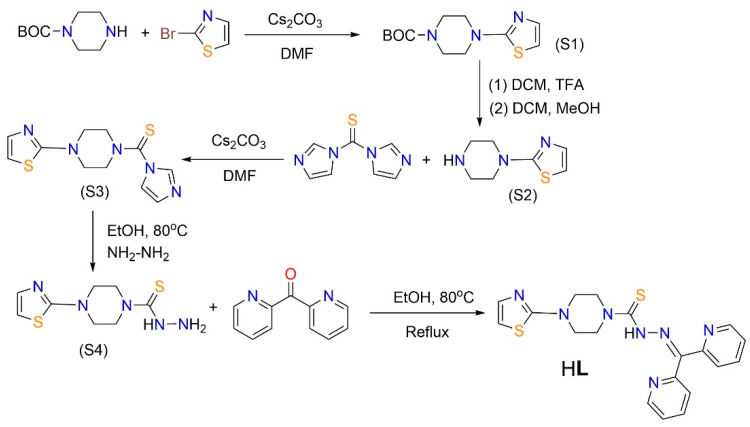
The five-step synthetic procedure for H**L**.

**Figure 1:**
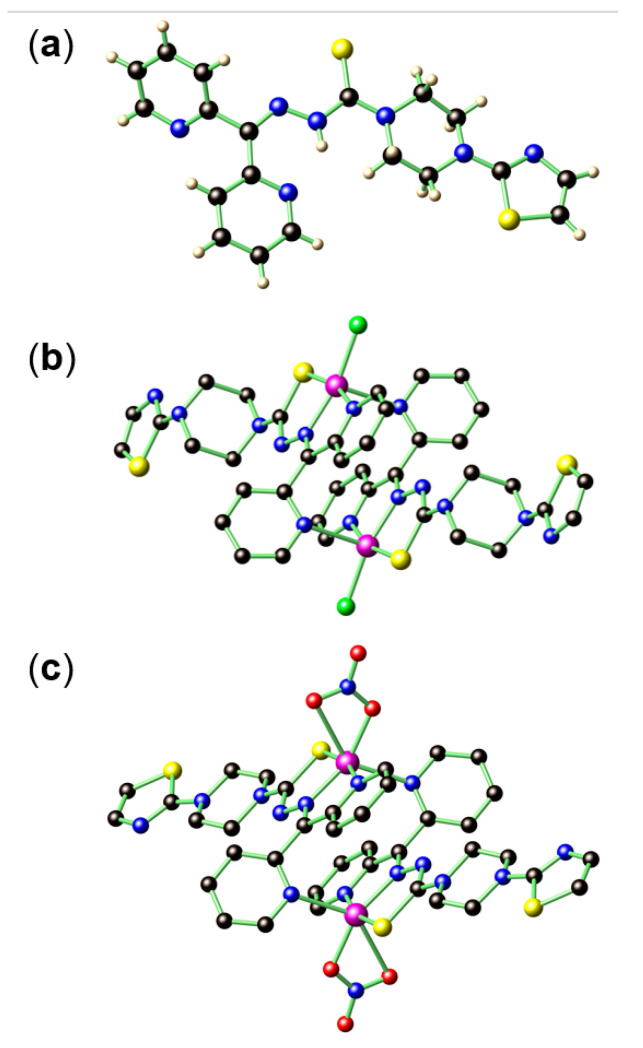
The single-crystal structure of H**L** (**a**), **1** (**b**), and **2** (**c**). In (**b**,**c**), the H atoms were also omitted for clarity. Color legend: Cu—dark magenta; Cl—green; S—yellow; O—red; N—blue; C—black; H—light pink.

The synthesis of ligand H**L** was achieved by a five-step protocol to give an overall yield of 9.3% (Scheme 2). The purity of H**L** was confirmed by microanalysis, ^1^H, and ^13^C nuclear magnetic resonance (NMR) spectroscopy (Figure S1). The assumed connectivity of H**L** was also unambiguously determined by single-crystal X-ray diffraction studies (Figure 1a).

**Scheme 3:**
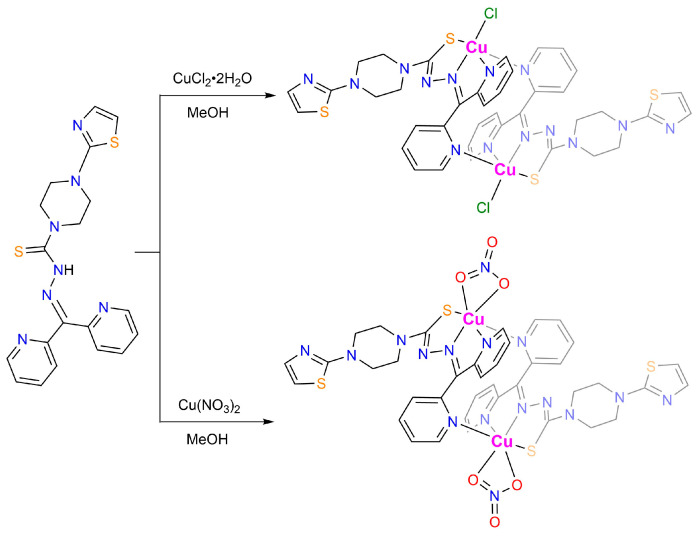
The synthetic procedure for **1** and **2** from H**L**.

**Figure 2:**
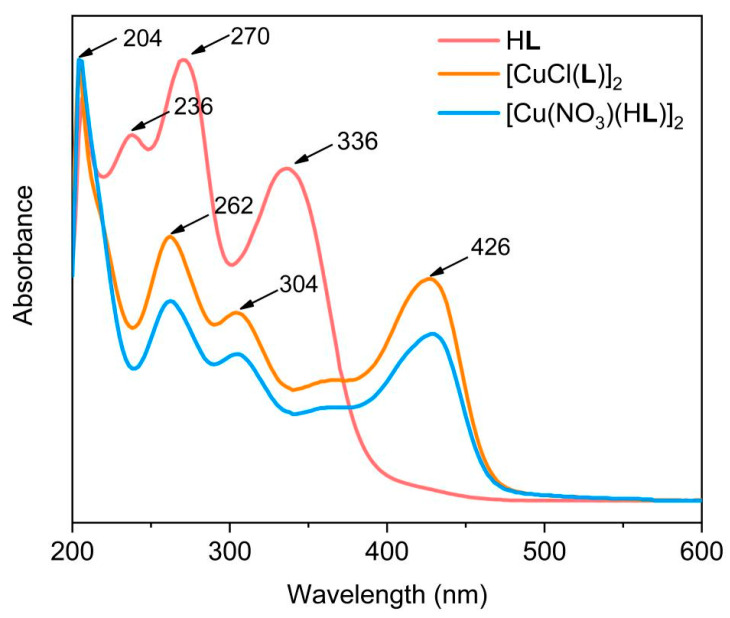
The UV-Vis spectra of H**L** (light red), **1** (light orange), and **2** (light blue) in MeOH solutions.

Coordination complexes of **1** and **2** were prepared accordingly by reacting H**L** and the respective Cu(II) sources under appropriate conditions (Scheme 3). The single-crystal X-ray structure data for **1** (Figure 1b) and **2** (Figure 1c) indicated that both compounds form dimeric structures in the solid state, enabled by the head-to-tail complementary Cu−N coordination. In **1**, each Cu^2+^ shows a square pyramidal coordination geometry, fulfilled by an S, one azomethinic N, one α-pyridyl N atom from an **L** ligand, and one Cl^−^ that defines the square plane, in addition to one pyridyl N atom from adjacent **L** ligand that occupies the apical position. A similar connectivity is also found in **2**, except that one Cl^−^ is replaced by a chelating NO_3_^−^, yielding a distorted octahedral coordination geometry for Cu^2+^.

Notably, the ligands in both **1** and **2** undergo a change of conjugation with the loss of hydrazinic proton and the conversion of C=S into a C−S bond. Such a structure variation is seen in a variety of transition metal complexes of α-pyridyl thiosemicarbazone chelators, such as Triapine complexes of Fe^3+^/Ga^3+^ [17,18], di-2-pyridyl ketone 4,4-dimethyl-3-thiosemicarbazone (HDp44mT) complexes of Fe^3+^ [19], as well as several Cu^2+^ complexes [20]. Bormio Nunes et al. also reported that, for Fe^3+^/Cu^2+^ complexes of COTI-2, the N−N single bond of the ligand is inherited in the final chelating complexes [16]. As a result of the C=S to C−S bond variation, the C=S bond distance in H**L** of 1.681(5) Å increased to 1.739(4) Å in **1** and 1.744(6) Å in **2** (Table S1). Nevertheless, the N−N distance in H**L** of 1.354(5) Å remained nearly unaltered as compared with the N=N distances in **1** (1.354(4) Å) and **2** (1.351(6) Å) due to the additional coordination of one N to Cu^2+^.

### 2.2. Spectroscopic Characterizations of H**L**, ***1***, and ***2***

**Figure 3:**
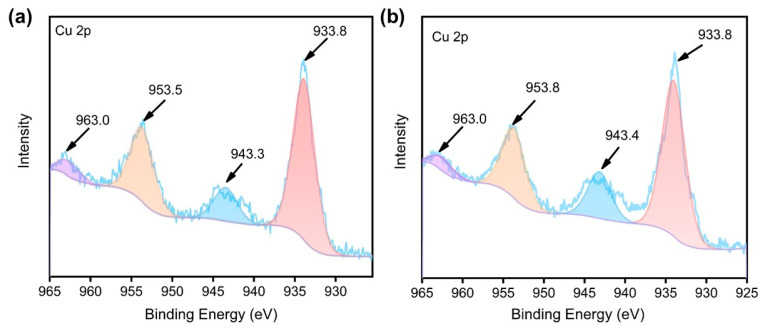
The Cu 2p XPS spectra of **1** (**a**) and **2** (**b**). For both **a** and **b**, peaks at 933.8 eV (light red)/953.5 eV (light orange) and 943.3 eV (light blue)/963.3 eV (light purple) are assigned as the spin-orbit splitting of Cu 2p_3/2_ and 2p_1/2_ of Cu^2+^/Cu^+^.

The energy-dispersive X-ray spectroscopy (EDS) reveal an atomic ratio of Cu:Cl:S of 2.0:2.2:4.2 in **1** and Cu:S of 2.0:4.6 in **2** (Figure S2), consistent with the derived ratio of Cu:Cl:S of 1.0:1.0:2.0 (**1**) and Cu:S of 1.0:2.0 (**2**) from the single-crystal data, and the elements Cu, S, N, and C are evenly distributed in their elemental mapping diagrams (Figure S2). The Fourier transform infrared (FT-IR) spectra show that upon coordination with Cu^2+^, the C=N bond vibration peaks at 1571 cm^−1^ in H**L** and shifts to 1593 cm^−1^ for both **1** and **2** (Figure S3). Meanwhile, bands at 850 cm^−1^ (**1**) and 856 cm^−1^ (**2**), which are assignable as C=S bond vibrations, also shift as compared with that of H**L** (869 cm^−1^) upon bonding with Cu^2+^ [21,22]. In the ultraviolet–visible (UV-Vis) spectroscopy (Figure 2), ligand-to-metal charge transfer (LMCT) bands in **1** and **2** are found at 426 nm as compared with H**L** [23]. Meanwhile, the ligand-centered UV-Vis absorption band of the H**L** undergoes a significant blue shift due to the conjugation change and charge transfer reactions. The LMCT leads to the partial reduction of Cu^2+^, which is favorable for Cu^+^-induced reactions, such as the ROS generation via the Fenton-like process [24]. The Cu 2p X-ray photoelectron spectroscopy (XPS) of both **1** and **2** revealed two sets of binding energies (Figure 3). Peaks at 933.8/953.5 eV (**1**) and 933.8/953.8 eV (**2**) are assignable as the spin-orbit splitting of Cu 2p_3/2_ and 2p_1/2_ of Cu^2+^ [25,26,27]. Two satellite peaks at 943.3/963.3 eV (**1**) and 943.4/963.0 eV (**2**) are also attributed to Cu^2+^ ions due to its d^9^ electronic configuration, which is susceptible to photoreduction to give a more stable d^10^ configuration. The liquid chromatography–mass spectrometry (LC-MS) for both **1** (Figure S4a) and **2** (Figure S4b) show peaks at 470.9 *m*/*z* and 488.8 *m*/*z*, assignable as the [Cu**L**]^+^ (molecular weight of 471.0) and [Cu**L**(H_2_O)]^+^ (molecular weight of 489.0), indicating that the dimeric structure of **1** and **2** presumably remain as monomers in solutions.

### 2.3. Hydroxyl Radical (•OH) Production

**Figure 4:**
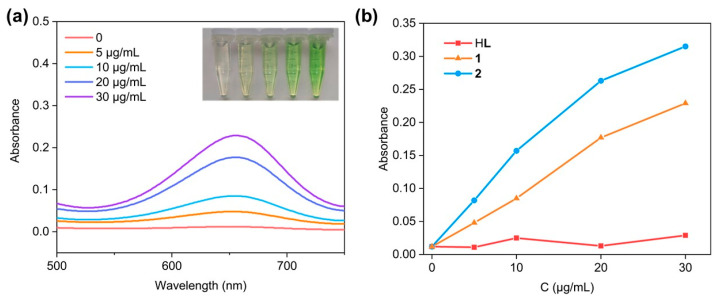
Change in UV-Vis absorption intensity in the range of 500–750 nm upon treatment with different concentrations of **1** in the presence of 100 μM H_2_O_2_ (**a**; inset: photographs showing color change upon introducing gradient concentrations of **1**). The intensity change at 652 nm (oxTMB) upon treating gradient concentrations of H**L**, **1**, and **2** with 100 μM H_2_O_2_ (**b**).

The generation of •OH from H_2_O_2_ by H**L**, **1**, and **2** was assayed by 3,3′,5,5′-tetramethylbenzidine (TMB) [12,28,29]. The colorless TMB can be quickly oxidized by •OH to give a characteristic blue charge-transfer complex oxTMB with intense absorption at 652 nm [30,31]. Different concentrations of H**L** (5, 10, 20, 30 μg/mL) or **1** and **2** containing equivalent H**L** were treated with (TMB) solution containing 100 μM H_2_O_2_, which is assumed as approximately the concentration reported to be present in tumor cells, or five times that in normal cells [32,33]. After 20 min of incubation with H**L**, there is no oxTMB found, as suggested by both the UV-Vis results and the direct observation of solution colors (Figure S5). In sharp contrast, both **1** and **2** can induce the generation of •OH under otherwise identical conditions in a concentration-dependent manner, with **2** further outperforming **1** (Figure 4 and Figure S5). In addition, the MeOH solutions of H**L**, **1**, and **2** at H**L**/**L** concentrations of 30 μg/mL remained stable upon keeping for 72 h (Figure S6), as evidenced by their unaltered UV-Vis absorption patterns throughout the experiments.

### 2.4. CCK-8 Assay for H**L**, ***1***, and ***2***

**Figure 5:**
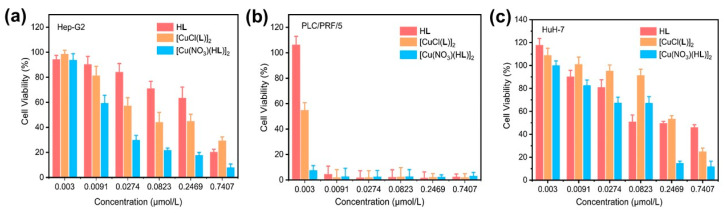
The viability of Hep-G2 (**a**), PLC/PRF/5 (**b**), and HuH-7 (**c**) cells when treated with gradient concentrations of H**L**, **1**, and **2** (n = 3).

The cytotoxicity of H**L**, **1**, and **2** against three hepatocellular carcinoma cell lines Hep-G2, PLC/PRF/5, and HuH-7 were evaluated by the cell counting kit-8 (CCK-8; APExBIO Technology, Houston, TX, USA) assay. As shown in Figure 5, all three complexes demonstrate superior cytotoxicity originating from the ligand, particularly for the PLC/PRF/5 cell line. Notably, **1** and **2** demonstrated higher cytotoxicity as compared with H**L**, presumably due to the additional ROS generation induced by the overexpressed H_2_O_2_ in the cancer cells. The IC_50_ values for H**L**, **1**, and **2** against PLC/PRF/5 were as low as 3.26 nmol/mL, 2.18 nmol/mL, and 2.54 × 10^−5^ nmol/mL, which is favorably lower than those against the Hep-G2 (78.83 nmol/mL for H**L**, 38.11 nmol/mL for **1**, and 16.86 nmol/mL for **2**) and HuH-7 (192.20 nmol/mL for H**L**, 176.60 nmol/mL for **1**, and 87.53 nmol/mL for **2**) cell lines (Table S2).

### 2.5. Intracellular ROS Generation by H**L**, ***1***, and ***2***

**Figure 6:**
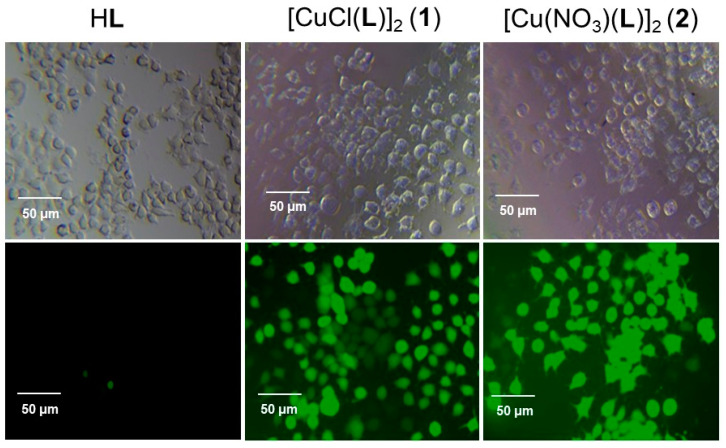
Comparison of the intracellular ROS generating ability of H**L**, **1**, and **2** in HuH-7 using DCFH-DA as a fluorescence probe.

Given the efficient •OH production in water induced by **1** and **2**, we next investigated in vitro ROS generation by **1** and **2** in the representative HuH-7 cell line using 2′,7′-dichlorodihydrofluorescein diacetate (DCFH-DA) as a fluorescence probe and compared it with that of H**L** [34,35]. The non-emissive DCFH-DA can be readily uptaken by the cell, where it is hydrolyzed into 2,7-dichlorodihydrofluorescein (DCFH) by cellular enzymes. Upon oxidation by ROS, DCFH can convert to 2,7-dichlorofluorescein (DCF), which gives bright green fluorescence [29,36]. As shown in Figure 6, H**L** alone produced negligible ROS. In sharp contrast, **1** and **2** can induce the formation of strong fluorescence, characterizing the effective generation of ROS in the HuH-7 cells.

### 2.6. In Vivo Pharmacokinetic Study of H**L**

**Figure 7:**
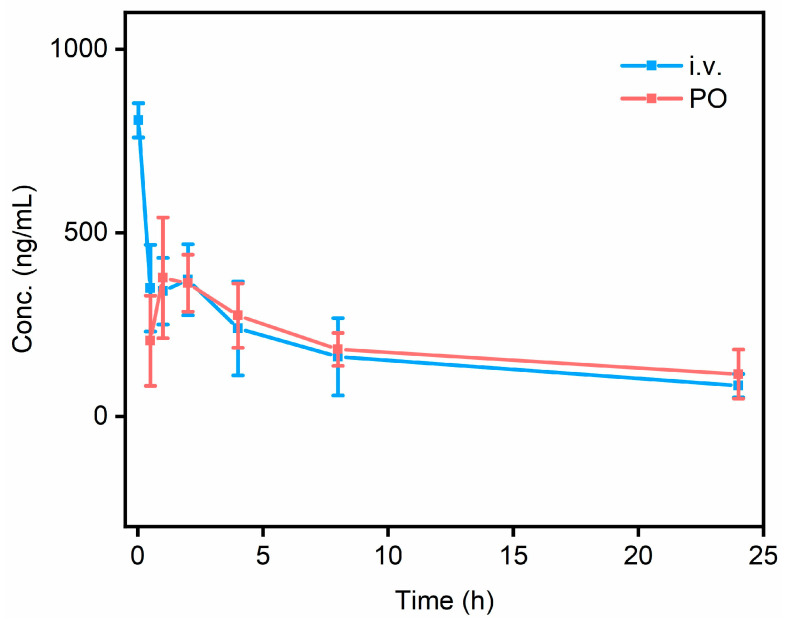
The time–concentration relationships of H**L** via i.v. (0.5 mg kg^−1^) and PO (30 mg kg^−1^) administrations.

For pharmacokinetic studies, CD^®^(SD) IGS rats (5−6 weeks old) were randomly divided into two groups (three for each group) for oral administration (per os, PO) or intravenous administration (i.v.) of H**L** with dosages of 30 mg kg^−1^ and 0.5 mg kg^−1^, respectively (Tables S3 and S4). The blood was then collected from the orbital sinus with a heparinized syringe at different time intervals (0.5, 1.0, 2.0, 4.0, 8.0, and 24 h for PO, and 0.03, 0.5, 1.0, 2.0, 4.0, 8.0, and 24 h for i.v.). High-performance liquid chromatography (HPLC) provided mean area under the curve (*AUC*_0-inf_) values of 10409.01 ± 5313.4 ng h mL^−1^ for PO and 5701.36 ± 2647.15 ng h mL^−1^ for i.v. H**L** achieved the largest *C*_max_ value of 425.93 ± 176.1 ng mL^−1^ at a *T*_max_ of 1.0 h for PO, while for i.v., the *C*_max_ value of 806.43 ± 46.66 ng mL^−1^ was immediately observed at *T*_max_ of 0.03 h, which is expected for i.v. administration. Notably, the PO administration exhibited a significantly longer half-life *t*_1/2_ of 21.61 ± 9.4 h, nearly doubled as compared with that of the i.v. administration of 11.88 ± 1.66 h (Figure 7), certifying that H**L** has a favorable pharmacokinetic via PO administration.

### 2.7. Toxicological Studies of H**L**

For toxicological studies, CD^®^(SD) IGS rats were randomly divided into six groups (three in each group; n = 3) for daily administration of H**L** (PO) with dosages of 5 mg kg^−1^ (12.2 μmol kg^−1^, groups 3 and 4) and 10 mg kg^−1^ (24.4 μmol kg^−1^, groups 5 and 6). Their weight and status were monitored and compared with the control group (groups 1 and 2) that was fed sterilizing solutions. As shown in Table S5, for groups 3 and 4, the rats remain alive throughout the experimental process with their weight constantly growing, similar to that found in groups 1 and 2 (Figure S7). In comparison, at a dosage of 10 mg kg^−1^, part of the rats (four of six) showed abnormal behaviors such as decreasing food uptake, cachexia, and lethargy on day 9, and these rats either died or were euthanized after drug suspension. The remaining two rats showed normal and stable behaviors throughout the experiments, with their final weights being comparable with those in control groups 1 and 2. This highlights that a 5 mg kg^−1^ drug dosage of H**L** by PO administration is safe for in vivo studies.

## 3. Materials and Methods

### 3.1. General

*tert*-butyl piperazine-1-carboxylate (BOC-PIP; ≥99%, Aladdin, Shanghai, China), 2-bromothiazole (98%, Crgent Biotech, Hangzhou, China), di-2-pyridyl ketone (98%, Crgent Biotech), NH_2_NH_2_ (≥95%, Adamas, Shanghai, China), Cu(NO_3_)_2_ (≥99%, Aladdin), CuO (≥97%, Aladdin), DMF (≥99%, Aladdin), CH_2_Cl_2_ (≥99%, Aladdin), trifluoroacetic acid (TFA; ≥99%, Aladdin), i-PrOH (≥99%, Aladdin), methyl *tert*-butyl ether (MTBE, ≥99%, Aladdin), NaHCO_3_ (>99%, Crgent Biotech), EtOH (≥95%), and Cs_2_CO_3_ (≥99%, Crgent Biotech) were commercially available and used without further purifications.

Hep-G2, PLC/PRF/5, and HuH-7 cell lines were purchased from the Shanghai Institute of Cell Biology, Chinese Academy of Sciences. Phosphate buffer solution (PBS) was purchased from Shanghai Basal Media Technologies Co., Ltd. (Shanghai, China). Cell culturing medium MEM (with NEAA + 10% FBS + 1% P/S), MEM (with NEAA + 1% P/S), DMEM (10% FBS + 1% P/S), McCoy’s 5A (10% FBS + 1% P/S), DMEM (1% P/S), and 0.25% trypsin solution (containing EDTA, dissolved in PBS) were purchased from Procell Life Science & Technology Co., Ltd. (Wuhan, China). The cell counting kit-8 (CCK-8) and the reactive oxygen species detection kit were from APExBIO (Houston, TX, USA) and Shanghai Beyotime Biotechnology Co., Ltd. (Shanghai, China), respectively.

^1^H nuclear magnetic resonance (NMR) spectra were obtained on a Varian UNITY plus-400/plus-600 NMR spectrometer (Varian, Inc., Palo Alto, CA, USA). Fourier transform infrared (FT-IR) spectra were measured on a Bruker VERTEX 70+HYPERION 2000 FT-IR spectrometer (Bruker AXS GmbH, Karlsruhe, Germany) using the attenuated total refraction (ATR) technique. Elemental analyses for C, H, and N were measured on a Carlo-Erba CHNO-S microanalyzer (Carlo Erba, Waltham, MA, USA). Ultraviolet–visible (UV-Vis) spectroscopy was obtained on a Varian Cary-50 UV–visible spectrophotometer (Varian, Inc., Palo Alto, CA, USA). X-ray photoelectron spectroscopy (XPS) was performed on an EXCALAB 250 XI X-ray photoelectron spectrometer (Thermo Scientific, Waltham, MA, USA). The high-performance liquid chromatography (HPLC) was carried out on an Agilent 1260 Infinity II Bio-SEC system (Agilent Technologies, Inc., Santa Clara, CA, USA). The liquid chromatography–mass spectrometry (LC-MS) was carried out on an Agilent 6130 Quadrupole LC/MS system (Agilent Technologies, Inc.) using MeOH as the mobile phase. The CCK-8 cytotoxicity assay was conducted on a multifunction microplate detector by recording the absorption at 450 nm using a TECAN M1000PRO microplate reader (Tecan, Zürich, Switzerland).

### 3.2. Synthetic Steps for H**L**

Ligand H**L** can be synthesized via a five-step process as indicated below.

Step 1. Synthesis of S1. *t*-Butyloxy carbonyl (BOC)-protected piperazine (BOC-PIP; 400.0 mg, 2.148 mmol) and 2-bromothiazole (529.0 mg, 3.221 mmol) were dissolved in DMF (4 mL) and stirred for 5 min. Cs_2_CO_3_ (1400 mg, 4.295 mmol) was added as a solid, and the mixture was heated overnight at 120 °C. The formed product was extracted from H_2_O using ethyl acetate to obtain S1 as a white solid. Yield (290 mg, 50% based on BOC-PIP); ^1^H NMR (400 MHz, CDCl_3_) δ 7.209 (s, 1H), 6.605 (s, 1H), 3.566 (s, 4H), 3.466 (s,4H), 1.483 (s, 9H); ^13^C NMR (101 MHz, CDCl_3_) δ 172.096, 154.601, 139.567, 107.814, 80.273, 48.456, 28.396.

Step 2. Synthesis of S2. Compound S1 (290.0 mg, 1.078 mmol) was dissolved in CH_2_Cl_2_ (4 mL) to obtain a light yellow-brown solution and trifluoroacetic acid (TFA, 12 mL) was subsequently added, and the solution quickly turned yellow upon stirring. The mixture was stirred for 2 h, and the solvent evaporated; DCM/MeOH = 2:1 and NaHCO_3_ were consequently introduced to obtain S2 as a light yellow powder. Yield (180 mg, 99% based on S1); ^1^H NMR (400 MHz, DMSO-*d*_6_) δ 9.845 (s, 1H), 7.360 (s, 1H), 7.088 (s, 1H), 5.039 (s, 7H), 3.843 (s, 4H), 3.257 (s, 4H); ^13^C NMR (101 MHz, DMSO-*d*_6_) δ 170.534, 133.871, 110.024, 46.268, 41.908.

Step 3. Synthesis of S3. Complex S2 (180.0 mg, 1.065 mmol) was added to CH_2_Cl_2_ (5 mL) to form a suspension, and Cs_2_CO_3_ (342.0 mg, 1.917 mmol) was introduced to give a yellow solution. The mixture was stirred at r.t. for 15 h, and the product was washed with H_2_O and extracted with CH_2_Cl_2_. The organic layers were combined and dried. The pure yellow compound of S3 can be isolated by chromatography using CH_2_Cl_2_/i-PrOH (25:1, *v*/*v*) as the eluent. Yield (270 mg, 91% based on S2); ^1^H NMR (400 MHz, CDCl_3_) δ 7.904 (s, 1H), 7.234 (s, 2H), 7.111 (s, 1H), 6.683 (s, 1H), 4.040 (s, 4H), 3.656 (s, 4H); ^13^C NMR (101 MHz, CDCl_3_) δ 179.348, 170.996, 139.720, 137.427, 130.303, 119.252, 108.941, 50.694, 48.032.

Step 4. Synthesis of S4. Compound S3 (270.0 mg, 0.968 mmol) was added to EtOH (4 mL) to form a suspension, and NH_2_NH_2_ (0.0063 mL, excess) was subsequently added. The mixture was heated to reflux at 80 °C to yield a white suspension of S4, which was purified by chromatography using CH_2_Cl_2_/MeOH (25:1, *v*/*v*) as the eluent. Yield (175 mg, 74% based on S3); ^1^H NMR (400 MHz, DMSO-*d*_6_) δ 9.225 (s, 1H), 7.189 (s, 1H), 6.881 (s, 1H), 4.802 (s, 2H), 3.882 (s, 4H), 3.417 (s, 4H); ^13^C NMR (101 MHz, DMSO-*d*_6_) δ 183.080, 171.525, 139.917, 108.901, 48.052, 46.913.

Step 5. Synthesis of H**L**. Compound S4 (40.0 mg, 0.165 mmol) was dissolved in EtOH (10 mL) and stirred for 10 min. Di-2-pyridyl ketone (34.0 mg, 0.181 mmol) was subsequently introduced, and the mixture was heated to reflux at 80 °C overnight. Upon cooling to r.t., the crude powder of H**L** was recrystallized with MeOH/MTBE (5:1, *v*/*v*) to give a pure yellow-green solid product of H**L**. The yield was 18.8 mg, 28%, based on S4. Single crystals of H**L** were grown by slow evaporation of a MeOH/DMSO (*v*/*v* = 1:1) solution of H**L**. Anal. calcd for C_19_H_19_N_7_S_2_: C 55.72, H 4.68, N 23.94; found: C 55.26; H 4.93; N, 23.49. IR (ATR, cm^−1^): 3053(w), 2842(w), 1572(m), 1519(vs), 1487(s), 1464(s), 1382(s), 1339(m), 1305(vs), 1279(vs), 1220(vs), 1204(vs), 1130(vs), 1104(s), 1048(s), 1016(vs), 995(vs), 962(s), 916(s), 898(w), 870(m), 858(w), 802(vs), 785(s), 762(m), 743(vs), 723(vs), 693(vs), 669(m), 656(m), 642(s), 618(s); ^1^H NMR (400 MHz, DMSO-*d*_6_) δ 14.64 (s, 1H), 8.89 (s, 1H), 8.61 (s, 1H), 7.98 (s, 3H), 7.60 (s, 3H), 7.20 (s, 1H), 6.89 (s, 1H), 4.16 (s, 4H), 3.32 (s, 4H); ^13^C NMR (101 MHz, DMSO-*d*_6_) δ 170.77, 148.40, 147.95, 139.47, 137.79, 137.30, 126.84, 124.64, 123.78, 108.38, 48.33, 47.45.

### 3.3. Synthesis and Characterization of [CuCl(**L**)]_2_ (***1***)

Method 1: H**L** (100 mg, 0.244 mmol) was introduced in 20 mL of MeOH to give a yellow suspension, and CuCl_2_•2H_2_O (42 mg, 0.246 mmol) in 10 mL of MeOH was subsequently introduced dropwise. The formed mixture was stirred at 40 °C for 8 h. The solvent was removed, and the formed brown powder was recrystallized in MeOH and Et_2_O to obtain the crystal of **1**.

Method 2: CuO (21.4 mg, 0.269 mmol) and HCl (19.6 mg, 0.537 mmol) were mixed in 3 mL of MeOH, and the mixture was stirred at 40 °C. With the gradual dissolution of CuO, the solution turned pale green. After 10 min, H**L** (100 mg, 0.244 mmol) was introduced, and the solution became dark brown. The mixture was stirred for an additional 2 h and then filtered, and it was washed with a mixture of MeOH/MTBE (1:1, *v*/*v*; MTBE = methyl tert-butyl ether) to give a brown powder of [CuCl(**L**)]_2_, which was dried under vacuo. Yield (220 mg, 89% based on H**L**); anal. calcd for C_38_H_40_Cl_2_Cu_2_N_14_S_4_: C 44.79, H 3.96, N 19.24; found: C 44.18, H 3.37, N 18.96; IR (ATR, cm^−1^): 3077(w), 2993(w), 2645(w), 1592(s), 1563(m), 1476(w), 1458(w), 1440(vs), 1418(s), 1383(s), 1364(vs), 1318(w), 1289(m), 1275(s), 1247(vs), 1193(m), 1169(w), 1130(s), 1089(w), 1058(w), 1012(s), 982(w), 966(w), 956(w), 923(s), 850(m), 812(m), 795(s), 772(w), 738(m), 709(m), 686(w), 664(s), 644(s), 612(m).

### 3.4. Synthesis and Characterization of [Cu(NO_3_)(**L**)]_2_ (***2***)

Cu(NO_3_)_2_ (50.5 mg, 0.269 mmol) and HL (100 mg, 0.244 mmol) were mixed in 3 mL of MeOH, and the mixture was stirred at 40 °C to obtain a dark brown solution; upon further stirring for 2 h, the solution turned dark green. The mixture was filtered and washed with a mixture of MeOH/MTBE (1:1, *v*/*v*; MTBE = methyl tert-butyl ether) to give a brown powder of 2, which was dried under vacuum. Single crystals were obtained by slow diffusion of Et_2_O into a MeOH solution of 2. Yield (235 mg, 90% based on HL); anal. calcd for C_38_H_36_Cu_2_N_16_O_6_S_4_: C 42.73, H 3.40, N 20.98; found: C 42.38, H 3.33, N 20.81; IR (ATR, cm^−1^): 3080(w), 2846(w), 1592(m), 1520(s), 1483(s), 1461(vs), 1424(vs), 1380(s), 1338(w), 1278(vs), 1239(vs), 1203(vs), 1140(vs), 1102(m), 1052(w), 1012(vs), 920(s), 897(w), 855(m), 819(m), 792(s), 744(m), 724(w), 700(m), 665(m), 615(m).

### 3.5. Single-Crystal X-ray Crystallography

Diffraction data for H**L**, **1**, and **2** were acquired either on a Bruker APEX II CCD X-ray diffractometer (Bruker AXS GmbH, Germany) using Mo-Kα (λ = 0.71073 Å) (H**L**) or Ga-Kα (λ = 1.34138 Å) irradiation (**1** and **2**). Refinement and reduction of the collected data were achieved using the program Bruker SAINT, and absorption corrections were performed using a multi-scan method [37]. All crystal structures were solved by direct methods and refined on *F*^2^ by full-matrix least-squares techniques with SHELXTL-2016 [38]. Crystallographic data for H**L**, **1**, and **2** have been deposited in the Cambridge Crystallographic Data Center (CCDC) as supplementary publication numbers 2,341,904 (H**L**), 2,341,905 (**1**), and 2,341,906 (**2**). These data can be obtained free of charge, either from the CCDC via www.ccdc.cam.ac.uk/data_request/cif (accessed on 21 March 2024) or from the Supplementary Materials. A summary of the key crystallographic data for H**L**, **1**, and **2** is listed in Table 1. Selected bond distances and angles were listed in Table S1.

### 3.6. In Vitro Cytotoxicity Evaluation by CCK-8 Assay

The HuH-7 cell line was cultured in DMEM + 10% FBS + 1% P/S and DMEM + 1% P/S. Cells grew as a monolayer and were detached upon confluence using trypsin (0.5% *w/v* in PBS). The cells were harvested from the cell culture medium by incubation in trypsin solution for 3 min, and they were then centrifuged with the supernatant and subsequently discarded. A 3 mL portion of serum-supplemented cell culture medium was added to neutralize any residual trypsin. The cells were re-suspended in serum-supplemented DMEM at a concentration of 5 × 10^4^ cells/mL. Cells were cultured at 37 °C and 5% CO_2_ for the CCK-8 studies. HuH-7 cells were seeded at a density of 2 × 10^4^ cells per well in 90 µL of culture medium (DMEM + 10% FBS + 1% P/S) and cultured for 24 h at 37 °C and 5% CO_2_ for attachment. The culture medium was then replaced by a serum-free medium (DMEM + 1% P/S) containing various concentrations of H**L**, **1**, and **2** (pharmaceuticals solubilized with 2 parts per thousand DMSO). All experiments were carried out with three replicates (n = 3), and the untreated cells served as the 100% cell viability control, while the cell-free medium (DMEM + 10% FBS + 1% P/S + CCK-8) served as the blank. HuH-7 cells were directly incubated for a period of 72 h. After incubation, 100 μL of culture medium (DMEM + 10% FBS + 1% P/S) and 10 μL of CCK-8 was introduced and cultured for an additional 2 h before spectrophotometric measurement at 450 nm on a microplate reader. The relative cell viability (%) related to control cells was calculated using the equation:

$$V\%=\frac{{\left[A\right]}_{experimental}-{\left[A\right]}_{blank}}{{\left[A\right]}_{control}-{\left[A\right]}_{blank}}\times 100\%$$

where V% is the percentage of cell viability, [A]_experimental_ is the absorbance of the wells culturing the treated cells, [A]_blank_ is the absorbance of the blank, and [A]_control_ is the absorbance of the wells culturing untreated cells. The cytotoxicity assessment for Hep-G2 and PLC/PRF/5 resembled that of HuH-7, except that the culturing media was different, viz. MEM (NEAA) + 10% FBS + 1% P/S and MEM (NEAA) + 1% P/S for both Hep-G2 and PLC/PRF/5.

### 3.7. Detection of Intracellular Reactive Oxygen Species

HuH-7 cells were introduced into a 12-well plate (4 × 10^5^ per well) in 1 mL of growing media (DMEM + 10% FBS + 1% P/S), and the cells were incubated at 37 °C under 5% CO_2_ for 24 h for attachment. The growing media were removed, and the wells were replenished with H**L**, **1**, and **2** in 1 mL of growing media (with 2 ppm of DMSO) with equivalent H**L** concentrations of 2 μg/mL. The cells were incubated for an additional 2 h, and then the culturing media was replaced with 10 μmol/mL DCFH-DA in DMEM + 1% P/S (1 mL), prepared by diluting (1:1000) a concentrated DCFH-DA following the supplied protocols. The cells were incubated for a further 20 min, and then the culturing media was removed. The cells were further rinsed with culturing media (DMEM + 1% P/S) three times to remove the residual DCFH-DA and were observed under a BD5000 inverted microscope to estimate and compare the ROS generation abilities of these materials.

### 3.8. Pharmacokinetics of H**L**

CD^®^(SD) IGS rats (5–6 weeks old) were purchased from Beijing Vital River Laboratory Animal Technology Co., Ltd. (Beijing, China). All animal experiments were conducted in accordance with the requirements of the Experimental Animal Welfare Ethics Committee of Transcenta Diagnostic Technology (Suzhou) Co., Ltd. (PZ-20211210001). For pharmacokinetic experiments, SD rats were randomly divided into two groups, with 3 for PO administration (30 mg kg^−1^) and i.v. administration (0.5 mg kg^−1^), respectively. Plasma was collected from the jugular vein with the presence of a heparinized syringe at different time intervals, viz. 0.5, 1.0, 2.0, 4.0, 8.0, and 24 h for PO and 0.03, 0.5, 1.0, 2.0, 4.0, 8.0, and 24 h for i.v. Plasma was first centrifuged at 3500 rpm for 10 min, and then 0.05 mL of the plasma was extracted; trifluoroacetic acid was used to acid precipitate protein, and NaOH was used to neutralize the solution. The mixture was diluted using MeCN: H_2_O (5:5 *v*/*v*). After 10 min of precipitating, supernatant fluids were collected by centrifugation at 10,000 rpm for 5 min and filtered with a syringe through a 0.22 μm hydrophilic membrane filter and measured using the HPLC method. For the HPLC assay, the analytical column was an Agilent ZORBAX SB C_18_ column (4.6 mm × 150 mm, 5 μm). The mobile phase was MeCN:H_2_O (5:5 *v*/*v*), the flow rate of the mobile phase was 0.8 mL min^−1^, and the UV detector was set at 245 nm.

### 3.9. Toxicological Studies

For toxicological studies, CD^®^(SD) IGS rats (5−6 weeks old) were randomly divided into six groups (three for each group) for the PO administration of H**L** with daily dosages of 5 mg kg^−1^ (12.2 μmol kg^−1^, groups 3 and 4) and 10 mg kg^−1^ (24.4 μmol kg^−1^, groups 5 and 6), and they were compared with the control group that was fed sterilizing solutions (groups 1 and 2, Table S5). The weight of the rats was continuously monitored over 27 days, and they were fed on day 1, 4, 8, 13, 16, 19, and 23. For groups 5 and 6, the drug dosage was suspended when abnormal behaviors were observed, including decreasing food uptake, cachexia, and lethargy.

## 4. Conclusions

In this work, we report that the thiosemicarbazone derivative of H**L** and its Cu(II) complexes **1** and **2** demonstrated superior anticancer performances against hepatocellular carcinoma cell lines Hep-G2, PLC/PRF/5, and HuH-7. For **1** and **2**, the involvement of ROS as catalyzed by the Fenton-like process is also obvious. Intriguingly, the pharmacokinetic studies revealed that H**L** can be successfully absorbed via PO administration, with a favorable half-life of 21.61 ± 9.4 h, almost double that of via i.v. administration, rendering H**L** as having promising clinical potential. We believe that the association of such a type of ligand with diverse metal ions may further equip the drug with additional beneficial features, such as the modulation of acute toxicity, toxicity via ROS, and on-demand metal supplements during the curation process.

## Figures and Tables

**Table 1:** A summary of the cell parameters and refinement results for H**L**, **1**, and **2**.

	HL	[CuCl(L)]_2_ (1)	[Cu(NO_3_)(L)]_2_ (2)
CCDC number	2341904	2341905	2341906
Formula	C_19_H_19_N_7_S_2_	C_38_H_40_Cl_2_Cu_2_N_14_S_4_	C_38_H_36_Cu_2_N_16_O_6_S_4_
Formula weight	409.53	1019.06	1068.15
Crystal system	Triclinic	Triclinic	Monoclinic
Space group	*P*-1	*P*-1	*P*2**_1_**/*n*
*a*/Å	10.4251(14)	9.342(2)	10.7226(16)
*b*/Å	10.7193(14)	10.714(2)	9.6646(15)
*c*/Å	11.0200(15)	11.378(3)	21.695(3)
*α*/°	105.347(5)	93.542(10)	90
*β*/°	103.115(5)	109.660(10)	104.187(2)
*γ*/°	117.474(5)	108.609(9)	90
*V*/Å^3^	962.0(2)	998.0(4)	2179.7(6)
*Z*	2	1	2
*D_c_*/(g cm^−3^)	1.414	1.696	1.627
*F*(000)	428	522	1092
*μ*/mm^−1^	0.298	8.151	1.235
Total reflns.	25863	16310	33191
Uniq. reflns.	3928	4037	4404
Observed reflns.	1855	3572	3194
Parameters	253	274	298
*R*_int_	0.1570	0.0818	0.0905
*R* ^a^	0.0882	0.0592	0.0677
*wR* ^b^	0.1738	0.1658	0.1470
*GOF* ^c^	1.029	1.079	1.048

^a^ *R* = Σ||*F*_o_| − |*F*_c_||/Σ|*F*_o_|. ^b^ *wR* = {Σ*w*(*F*_o_^2^ − *F*_c_^2^)^2^/Σ*w*(*F*_o_^2^)^2^}^1/2^. ^c^ *GOF* = {Σ*w*((*F*_o_^2^ − *F*_c_^2^)^2^)]/(*n − p*)}^1/2^, where *n* = number of reflections and *p* = total number of parameters refined.

## Data Availability

The data that support the findings of this study are available from the corresponding authors upon request.

## Supplementary Materials

The following supporting information can be downloaded at: https://www.mdpi.com/article/10.3390/molecules29163832/s1, additional figures on NMR, EDS, FT-IR, LC-MS, UV-Vis, CD^®^(SD) IGS Rats status record, and tables on bond distance and angles, IC_50_ values, pharmacokinetic data for i.v. and PO administration, CD^®^(SD) IGS Rats weight record.
