# Supplementary material for: Thiazole Functionalization of Thiosemicarbazone for Cu(II) Complexation: Moving toward Highly Efficient Anticancer Drugs with Promising Oral Bioavailability
Source: Molecules. 2024 Aug 13;29(16):3832. doi: 10.3390/molecules29163832 (PMC11357102; doi:10.3390/molecules29163832)
Supplement: Supplementary file 1 [file molecules-29-03832-s001.zip › molecules-3137863-supplementary.pdf]

*Supplementary Material for*

# Thiazole Functionalization of Thiosemicarbazone for Cu(II) Complexation: Moving toward Highly Efficient Anticancer Drugs with Promising Oral Bioavailability

**Song-Yu Luo [1,†], Chun-Mei Zeng [1,†], Ping Xu [2], Ye Ning [1], Meng-Lin Dong [1], Wen-Hua Zhang [1,*] and Guangliang Yu [2,*]**

1 College of Chemistry, Chemical Engineering, and Materials Science, Soochow University, Suzhou 215123, China

2 Suzhou Degen Bio-Medical Co., Ltd., No. 1 Huayun Road, Suzhou Industrial Park, Suzhou 215000, China

* Correspondence: whzhang@suda.edu.cn (W.-H.Z.); yuguangliang@degrontech.onaliyun.com (G.Y.)

† These authors contributed equally to this work.

## Table of Contents

**Figure S1.** $^{1}H$ (a) and $^{13}C$ (b) NMR spectrum of H**L** in DMSO-$d_6$. ........ 3

**Figure S2.** The EDS elemental ratios of **1** (a) and **2** (b) and their respective elemental mapping diagrams. ........ 4

**Figure S3.** The FT-IR of H**L** (light red), **1** (light orange), and **2** (light blue) ........ 5

**Figure S4.** The liquid chromatography-mass spectrometry (LC-MS) for **1** (a) and **2** (b) using MeOH as the mobile phase. ........ 6

**Figure S5.** Change of UV-Vis absorption intensity in the range of 500–750 nm (peaked at 652 nm; oxTMB) upon treatment with different concentrations of H**L** (a) and **2** (b) in the presence of 100 μM $H_2O_2$ (insets: photographs showing color change upon introducing gradient concentrations of H**L** (a) and **2** (b)). ........ 6

**Figure S6.** The UV-Vis spectrum of H**L** (a), **1** (b), and **2** (c) in MeOH solutions upon keeping for 24 and 72 h, demonstrating the stability of these species in solutions. ........ 7

**Figure S7.** The record of CD®(SD) IGS Rats as ministered orally with sterilizing solution (groups 1, 2), 5 mg $kg^{-1}$ (groups 3, 4), and 10 mg $kg^{-1}$ (groups 5, 6) of H**L**. Color codes: green: normal; light blue: died; pink: euthanized; cyan: drug suspended ........ 8

**Table S1.** Selected bond lengths (Å) and bond angles (°) for H**L**, **1** and **2**. ........ 9

**Table S2**. The half-maximal inhibitory concentrations ($IC_{50}$) of H**L**, **1**, and **2** against different cell lines. ........ 14

**Table S3**. The pharmacokinetic data for H**L** via i.v. administration with dosages of 0.5 mg $kg^{-1}$ in ICR mice (n = 3). ........ 15

**Table S4**. The pharmacokinetic data for H**L** via PO administration with dosages of 30 mg $kg^{-1}$ in ICR mice (n = 3). ........ 16

**Table S5**. The weight record of CD®(SD) IGS Rats as ministered orally with sterilizing solution (groups 1, 2), 5 mg $kg^{-1}$ (groups 3, 4), and 10 mg $kg^{-1}$ (groups 5, 6) of H**L**. ........ 17

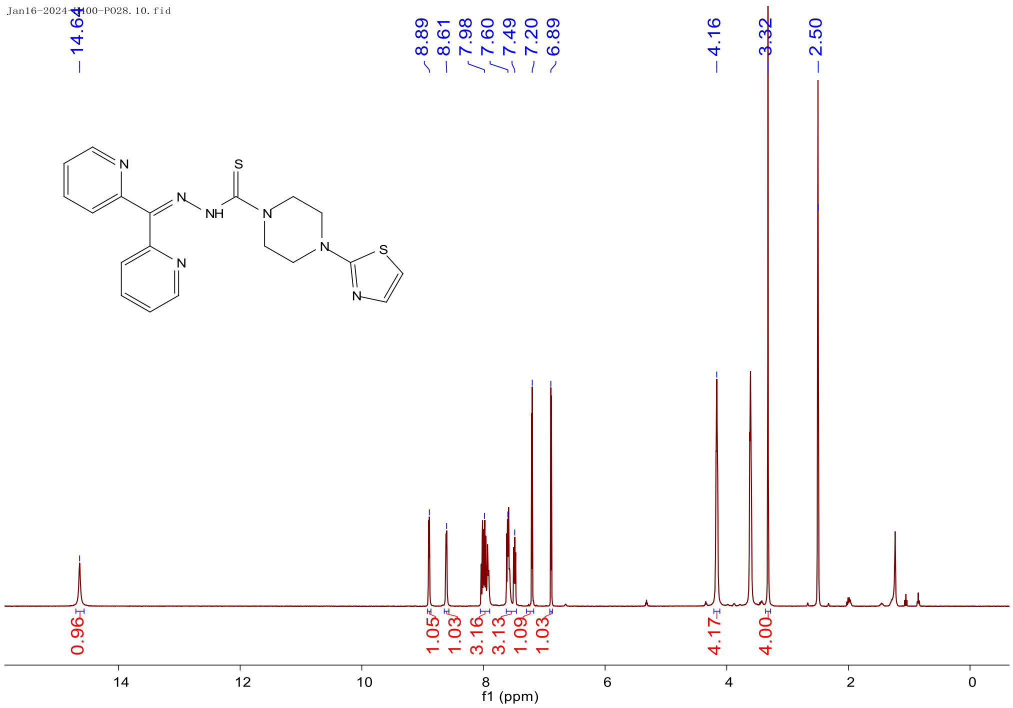


(a)

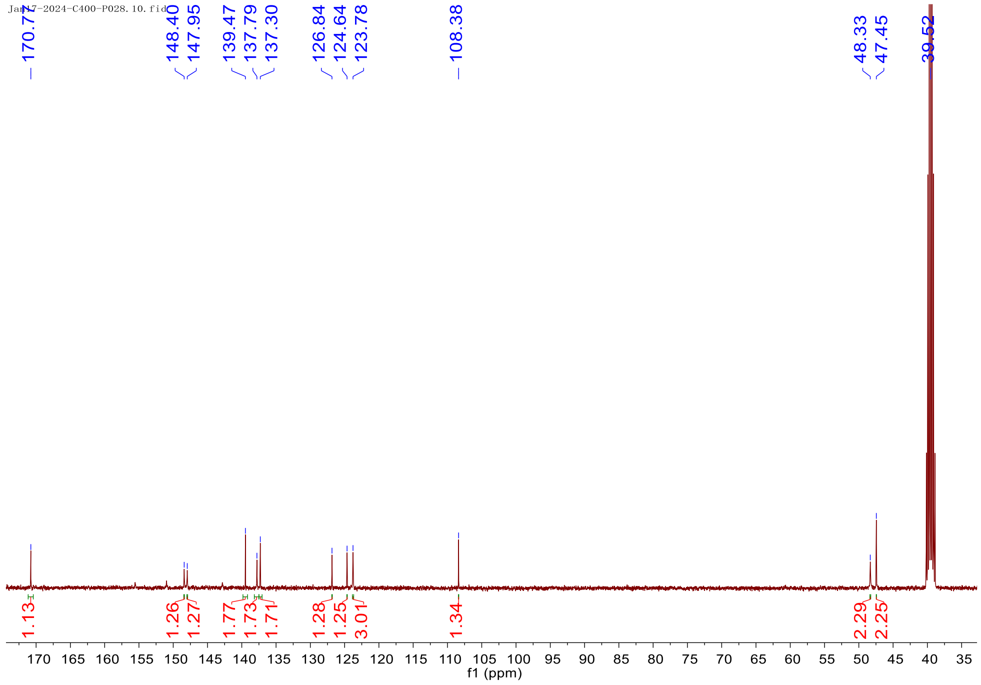


(b)

**Figure S1.** $^{1}H$ (a) and $^{13}C$ (b) NMR spectrum of H**L** in DMSO-$d_6$.

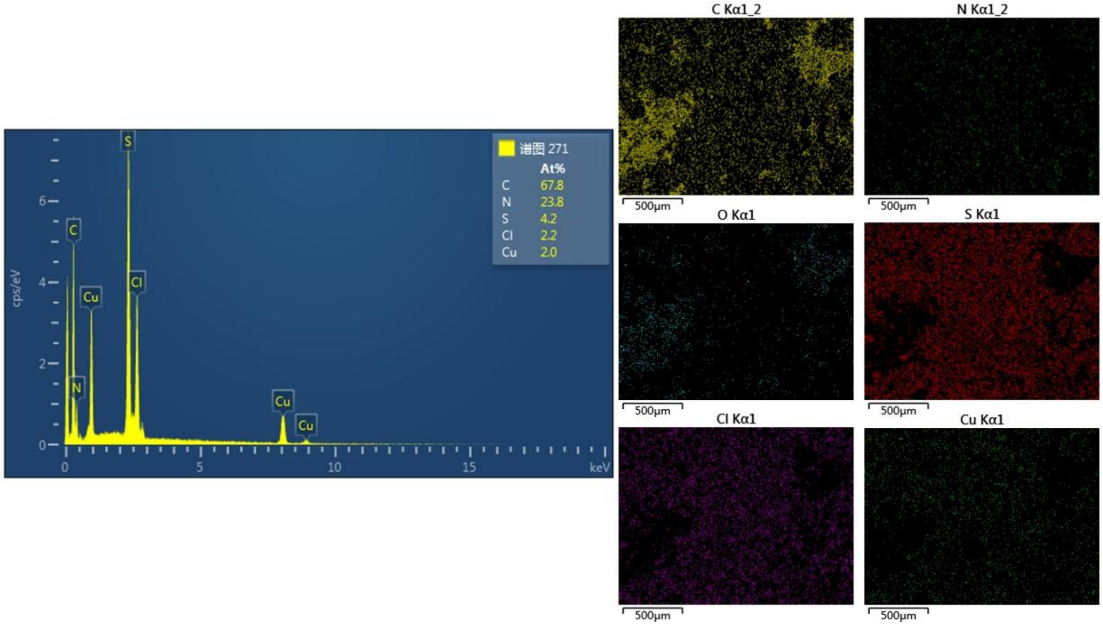


(a)

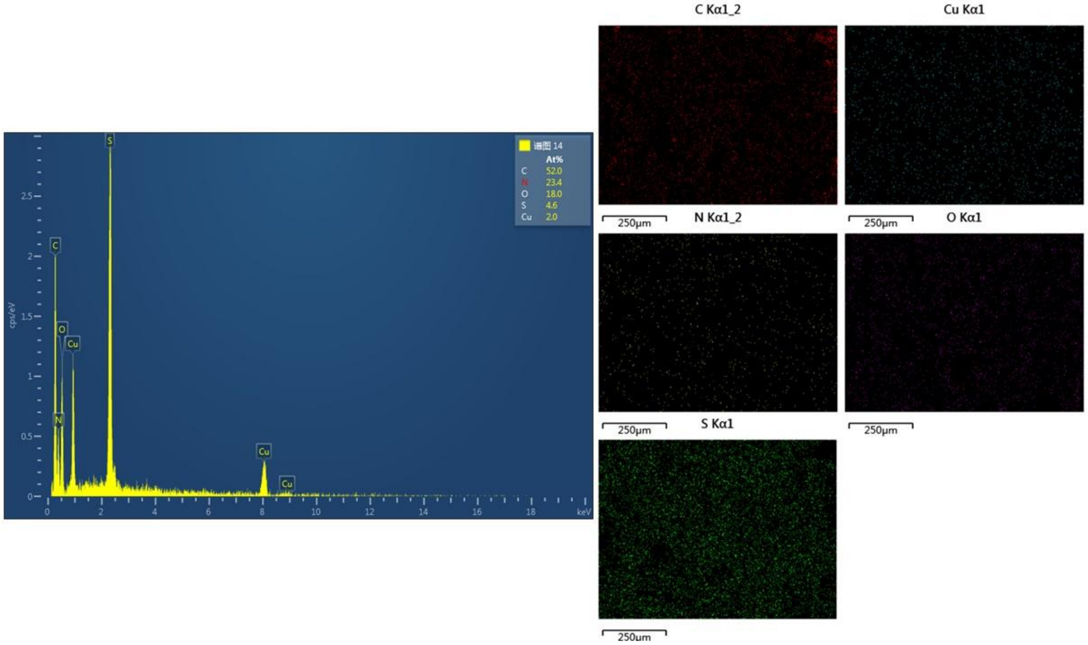


(b)

**Figure S2.** The EDS elemental ratios of **1** (a) and **2** (b) and their respective elemental mapping diagrams.

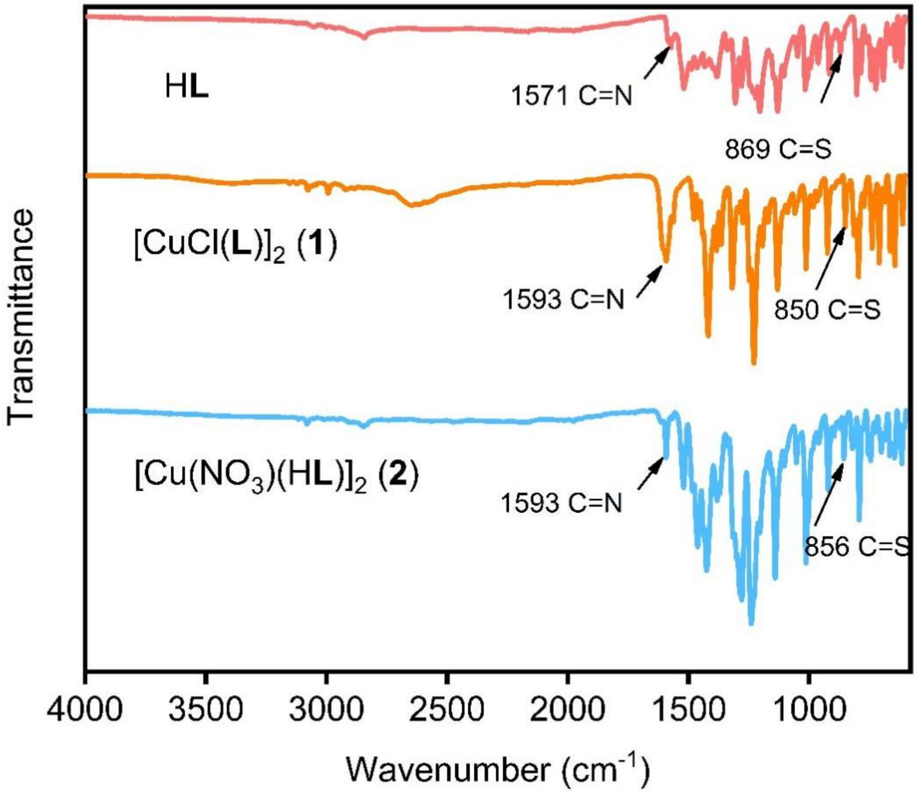


**Figure S3.** The FT-IR of H**L** (light red), **1** (light orange), and **2** (light blue).

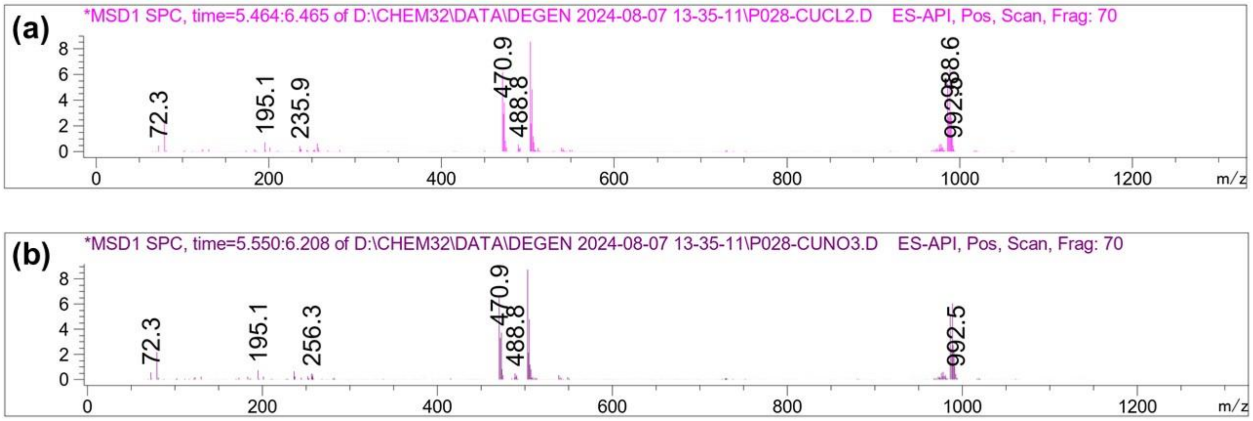


**Figure S4.** The liquid chromatography-mass spectrometry (LC-MS) for **1** (a) and **2** (b) using MeOH as the mobile phase.

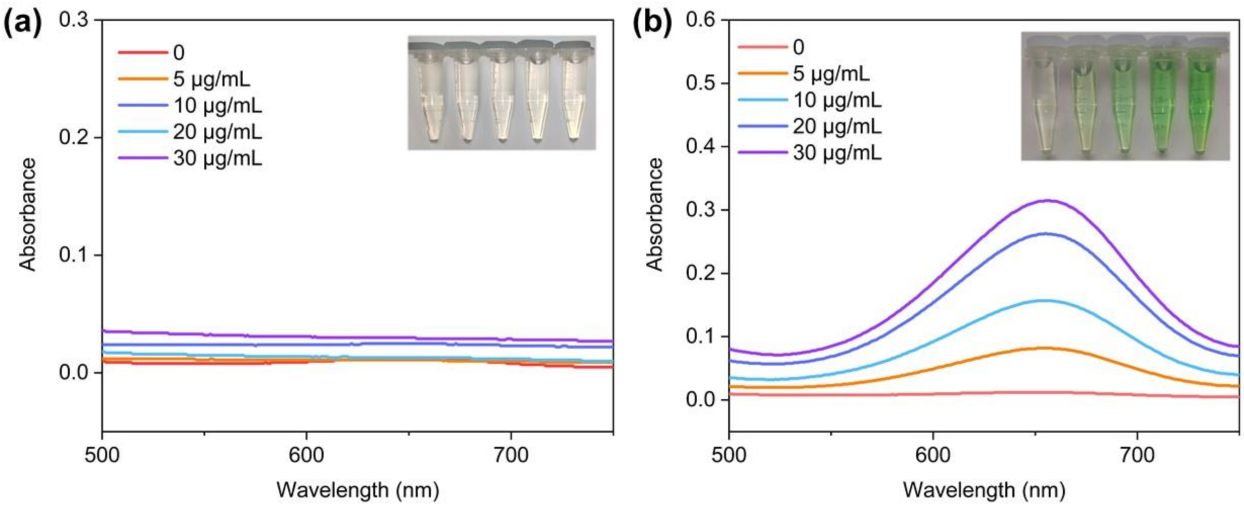


**Figure S5.** Change of UV-Vis absorption intensity in the range of 500−750 nm (peaked at 652 nm; oxTMB) upon treatment with different concentrations of H**L** (a) and **2** (b) in the presence of 100 μM $H_2O_2$ (insets: photographs showing color change upon introducing gradient concentrations of H**L** (a) and **2** (b)).

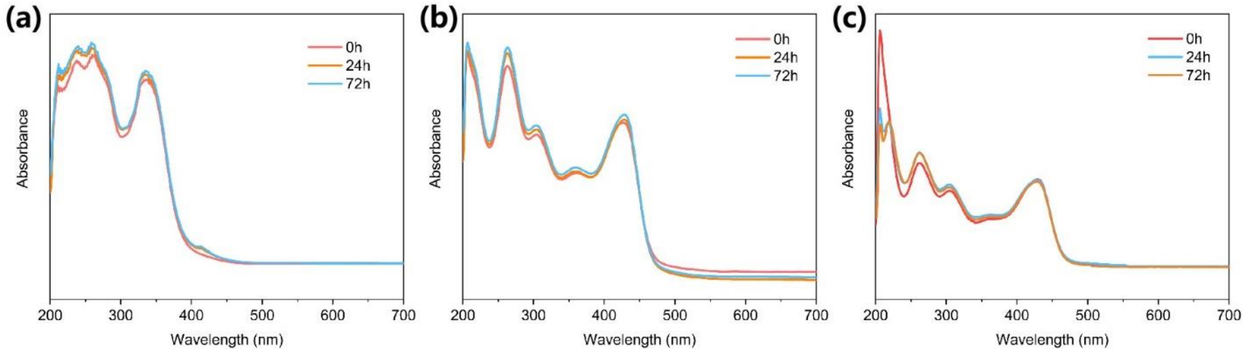


**Figure S6.** The UV-Vis spectrum of H**L** (a), **1** (b), and **2** (c) in MeOH solutions upon keeping for 24 and 72 h, demonstrating the stability of these species in solutions.

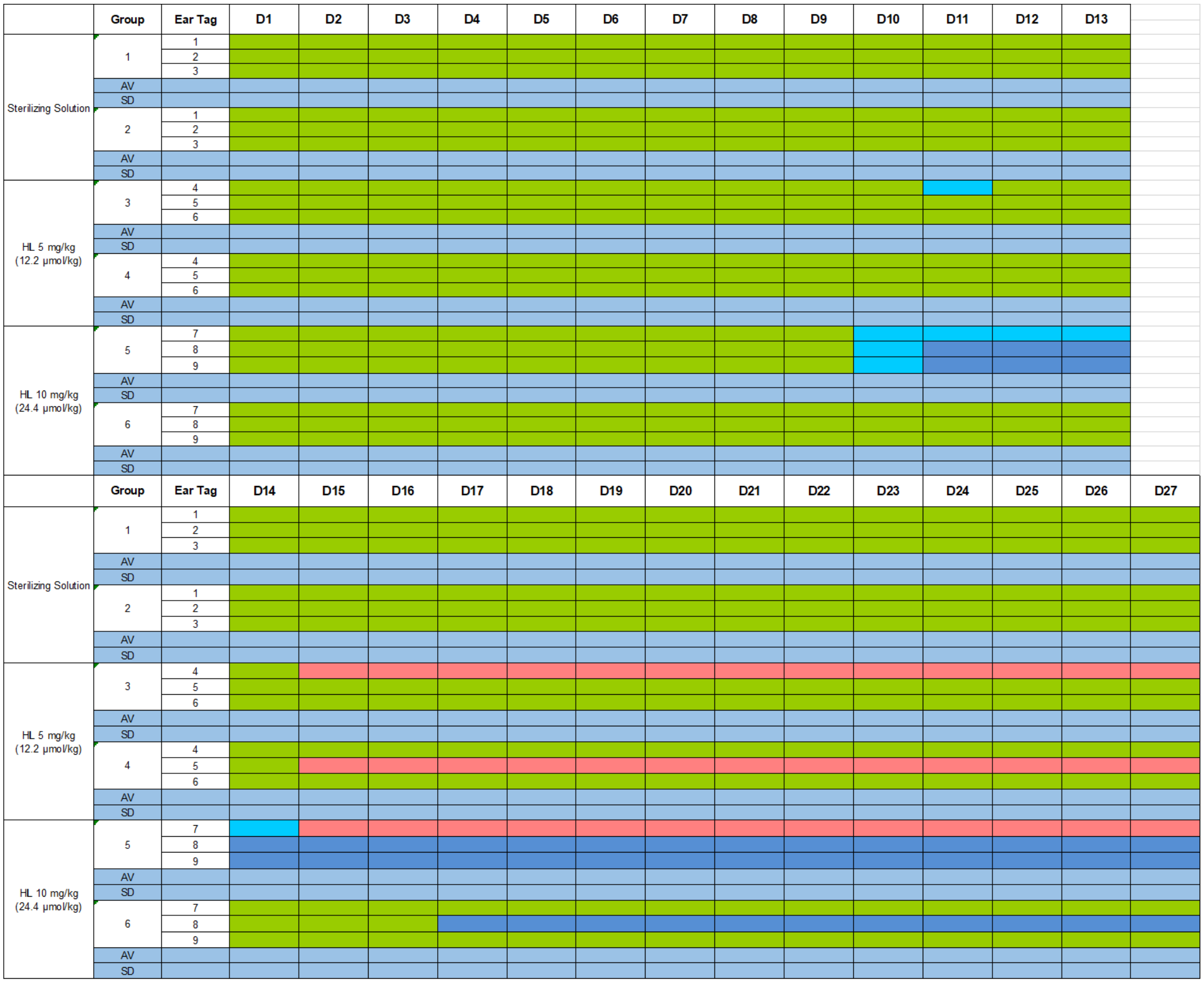


**Figure S7.** The record of CD®(SD) IGS Rats as ministered orally with sterilizing solution (groups 1, 2), 5 mg kg$^{-1}$ (groups 3, 4), and 10 mg kg$^{-1}$ (groups 5, 6) of H**L**. Color codes: green: normal; light blue: died; pink: euthanized; cyan: drug suspended.

**Table S1.** Selected bond lengths (Å) and bond angles (°) for H**L**, **1** and **2**.

| Compound H**L** | | | |
|---|---|---|---|
| S(1)-C(1) | 1.706(6) | S(1)-C(3) | 1.731(5) |
| S(2)-C(8) | 1.681(5) | N(1)-C(3) | 1.314(6) |
| N(1)-C(2) | 1.385(7) | N(2)-C(3) | 1.363(6) |
| N(2)-C(7) | 1.401(7) | N(2)-C(4) | 1.430(6) |
| N(3)-C(8) | 1.356(6) | N(3)-C(6) | 1.453(6) |
| N(3)-C(5) | 1.467(6) | N(4)-N(5) | 1.354(5) |
| N(4)-C(8) | 1.363(6) | C(4)-C(5) | 1.462(7) |
| N(5)-C(9) | 1.308(6) | N(6)-C(10) | 1.325(6) |
| N(6)-C(14) | 1.352(7) | N(7)-C(19) | 1.334(6) |
| N(7)-C(15) | 1.350(6) | C(1)-C(2) | 1.317(8) |
| C(9)-C(15) | 1.473(6) | C(6)-C(7) | 1.414(8) |
| C(10)-C(11) | 1.364(7) | C(11)-C(12) | 1.372(7) |
| C(9)-C(10) | 1.501(6) | C(12)-C(13) | 1.348(8) |
| C(17)-C(18) | 1.383(8) | C(13)-C(14) | 1.352(8) |
| C(15)-C(16) | 1.386(7) | C(16)-C(17) | 1.390(7) |
| C(18)-C(19) | 1.371(7) | | |
| | | | |
| C(1)-S(1)-C(3) | 89.2(3) | C(3)-N(1)-C(2) | 108.6(5) |
| C(3)-N(2)-C(7) | 121.7(4) | C(3)-N(2)-C(4) | 119.3(4) |
| C(7)-N(2)-C(4) | 113.2(4) | C(8)-N(3)-C(6) | 123.7(4) |
| C(8)-N(3)-C(5) | 119.5(4) | C(6)-N(3)-C(5) | 114.7(4) |
| N(5)-N(4)-C(8) | 120.5(4) | C(9)-N(5)-N(4) | 118.4(4) |
| C(10)-N(6)-C(14) | 115.9(5) | C(19)-N(7)-C(15) | 118.8(4) |
| C(2)-C(1)-S(1) | 110.2(4) | C(1)-C(2)-N(1) | 117.5(5) |
| N(1)-C(3)-N(2) | 124.1(5) | N(1)-C(3)-S(1) | 114.5(4) |
| N(2)-C(3)-S(1) | 121.4(4) | N(2)-C(4)-C(5) | 112.8(5) |
| C(4)-C(5)-N(3) | 113.7(5) | C(7)-C(6)-N(3) | 115.6(5) |
| N(3)-C(8)-N(4) | 113.7(4) | N(2)-C(7)-C(6) | 119.4(6) |
| N(3)-C(8)-S(2) | 124.0(4) | N(4)-C(8)-S(2) | 122.3(4) |
| N(5)-C(9)-C(15) | 128.5(4) | N(5)-C(9)-C(10) | 110.6(4) |
| C(15)-C(9)-C(10) | 121.0(4) | N(6)-C(10)-C(11) | 122.0(5) |
| N(6)-C(10)-C(9) | 116.4(4) | C(11)-C(10)-C(9) | 121.5(5) |

Table S1 Continued

| | | | |
|---|---|---|---|
| C(10)-C(11)-C(12) | 120.4(5) | C(13)-C(12)-C(11) | 118.6(6) |
| C(12)-C(13)-C(14) | 118.0(5) | C(13)-C(14)-N(6) | 125.0(6) |
| N(7)-C(15)-C(16) | 120.7(4) | N(7)-C(15)-C(9) | 117.0(4) |
| C(16)-C(15)-C(9) | 122.2(4) | C(15)-C(16)-C(17) | 119.7(5) |
| C(18)-C(17)-C(16) | 119.0(5) | C(19)-C(18)-C(17) | 118.1(5) |
| N(7)-C(19)-C(18) | 123.7(5) | | |

Compound **1**

| | | | |
|---|---|---|---|
| Cu(1)-N(3) | 1.989(3) | Cu(1)-N(2) | 2.016(3) |
| Cu(1)-Cl | 2.2435(10) | Cu(1)-S(2) | 2.2518(10) |
| N(1)-C(1) | 1.344(5) | N(1)-C(5) | 1.353(4) |
| N(2)-C(11) | 1.336(4) | N(2)-C(7) | 1.350(4) |
| N(3)-C(6) | 1.306(4) | N(3)-N(4) | 1.354(4) |
| N(4)-C(12) | 1.343(4) | N(5)-C(12) | 1.352(5) |
| N(5)-C(15) | 1.472(4) | N(5)-C(13) | 1.474(4) |
| N(6)-C(17) | 1.378(5) | N(6)-C(14) | 1.444(5) |
| N(6)-C(16) | 1.476(4) | N(7)-C(18) | 1.361(5) |
| N(7)-C(17) | 1.373(5) | C(1)-C(2) | 1.387(5) |
| C(6)-C(7) | 1.477(5) | C(2)-C(3) | 1.393(5) |
| C(8)-C(9) | 1.390(5) | C(3)-C(4) | 1.377(5) |
| C(9)-C(10) | 1.376(5) | C(4)-C(5) | 1.396(5) |
| C(10)-C(11) | 1.388(5) | C(5)-C(6) | 1.482(5) |
| C(12)-S(2) | 1.739(4) | C(7)-C(8) | 1.395(5) |
| C(13)-C(14) | 1.536(5) | C(15)-C(16) | 1.529(5) |
| C(17)-S(1) | 1.730(4) | C(18)-C(19) | 1.336(6) |
| C(19)-S(1) | 1.713(4) | | |

| | | | |
|---|---|---|---|
| N(3)-Cu(1)-N(2) | 80.60(12) | N(6)-C(14)-C(13) | 111.4(3) |
| N(3)-Cu(1)-Cl | 165.09(9) | N(2)-Cu(1)-Cl | 97.99(9) |
| N(3)-Cu(1)-S(2) | 83.91(9) | N(2)-Cu(1)-S(2) | 163.71(9) |
| Cl-Cu(1)-S(2) | 95.66(4) | C(1)-N(1)-C(5) | 116.7(3) |
| C(11)-N(2)-C(7) | 119.5(3) | C(11)-N(2)-Cu(1) | 126.7(2) |
| C(7)-N(2)-Cu(1) | 113.5(2) | C(6)-N(3)-N(4) | 120.4(3) |
| C(6)-N(3)-Cu(1) | 116.1(2) | N(4)-N(3)-Cu(1) | 123.4(2) |
| C(12)-N(4)-N(3) | 111.9(3) | C(12)-N(5)-C(15) | 120.6(3) |

Table S1 Continued

| | | | |
|---|---|---|---|
| C(12)-N(5)-C(13) | 122.1(3) | C(15)-N(5)-C(13) | 111.3(3) |
| C(17)-N(6)-C(14) | 116.9(3) | C(17)-N(6)-C(16) | 119.9(3) |
| C(14)-N(6)-C(16) | 112.0(3) | C(18)-N(7)-C(17) | 108.0(3) |
| N(1)-C(1)-C(2) | 124.3(3) | C(3)-C(4)-C(5) | 119.3(3) |
| C(4)-C(3)-C(2) | 119.0(3) | C(1)-C(2)-C(3) | 117.9(3) |
| N(1)-C(5)-C(4) | 122.6(3) | N(1)-C(5)-C(6) | 116.4(3) |
| C(4)-C(5)-C(6) | 121.0(3) | N(3)-C(6)-C(7) | 114.8(3) |
| N(3)-C(6)-C(5) | 123.9(3) | C(7)-C(6)-C(5) | 121.1(3) |
| N(2)-C(7)-C(8) | 121.0(3) | N(2)-C(7)-C(6) | 114.7(3) |
| C(8)-C(7)-C(6) | 124.3(3) | C(9)-C(8)-C(7) | 118.7(3) |
| C(10)-C(9)-C(8) | 120.1(3) | C(9)-C(10)-C(11) | 118.0(3) |
| N(2)-C(11)-C(10) | 122.6(3) | N(4)-C(12)-N(5) | 116.7(3) |
| N(4)-C(12)-S(2) | 124.6(3) | N(5)-C(12)-S(2) | 118.7(3) |
| N(5)-C(13)-C(14) | 107.9(3) | N(5)-C(15)-C(16) | 109.1(3) |
| N(6)-C(16)-C(15) | 111.1(3) | C(18)-C(19)-S(1) | 110.2(3) |
| N(7)-C(17)-N(6) | 124.8(3) | N(7)-C(17)-S(1) | 113.9(3) |
| N(6)-C(17)-S(1) | 121.1(3) | C(19)-C(18)-N(7) | 118.4(4) |
| C(12)-S(2)-Cu(1) | 95.73(12) | C(19)-S(1)-C(17) | 89.5(2) |

Compound **2**

| | | | |
|---|---|---|---|
| Cu(1)-N(5) | 1.961(4) | Cu(1)-N(6) | 2.017(4) |
| Cu(1)-O(1) | 2.024(4) | Cu(1)-S(2) | 2.2733(16) |
| Cu(1)-N(7)#1 | 2.357(5) | S(1)-C(1) | 1.724(7) |
| S(1)-C(3) | 1.748(6) | S(2)-C(8) | 1.744(6) |
| O(1)-N(8) | 1.281(6) | O(2)-N(8) | 1.226(6) |
| O(3)-N(8) | 1.235(7) | N(1)-C(3) | 1.300(8) |
| N(1)-C(2) | 1.379(8) | N(2)-C(3) | 1.368(7) |
| N(2)-C(7) | 1.428(8) | N(2)-C(4) | 1.444(8) |
| N(3)-C(8) | 1.353(7) | N(3)-C(6) | 1.451(8) |
| N(3)-C(5) | 1.469(8) | N(4)-C(8) | 1.323(7) |
| N(4)-N(5) | 1.351(6) | N(5)-C(9) | 1.301(7) |
| N(6)-C(14) | 1.333(7) | N(6)-C(10) | 1.347(7) |
| N(7)-C(15) | 1.335(7) | N(7)-C(19) | 1.341(7) |
| C(1)-C(2) | 1.332(10) | C(4)-C(5) | 1.402(10) |
| C(6)-C(7) | 1.393(11) | C(9)-C(10) | 1.468(7) |

Table S1 Continued

| | | | |
|---|---|---|---|
| C(9)-C(15) | 1.491(7) | C(10)-C(11) | 1.396(8) |
| C(11)-C(12) | 1.389(8) | C(12)-C(13) | 1.368(9) |
| C(13)-C(14) | 1.382(9) | C(15)-C(16) | 1.380(8) |
| C(16)-C(17) | 1.376(8) | C(17)-C(18) | 1.377(9) |
| C(18)-C(19) | 1.373(9) | | |
| | | | |
| N(5)-Cu(1)-N(6) | 80.90(18) | N(7)-C(19)-C(18) | 123.5(6) |
| N(5)-Cu(1)-O(1) | 156.10(17) | N(6)-Cu(1)-O(1) | 93.88(17) |
| N(5)-Cu(1)-S(2) | 84.14(13) | N(6)-Cu(1)-S(2) | 164.95(14) |
| O(1)-Cu(1)-S(2) | 99.18(12) | N(5)-Cu(1)-N(7)#1 | 116.76(17) |
| N(6)-Cu(1)-N(7)#1 | 89.60(18) | O(1)-Cu(1)-N(7)#1 | 86.32(16) |
| S(2)-Cu(1)-N(7)#1 | 98.63(12) | C(1)-S(1)-C(3) | 88.2(3) |
| C(8)-S(2)-Cu(1) | 94.53(19) | N(8)-O(1)-Cu(1) | 109.3(3) |
| C(3)-N(1)-C(2) | 109.1(5) | C(3)-N(2)-C(7) | 121.5(5) |
| C(3)-N(2)-C(4) | 118.7(5) | C(7)-N(2)-C(4) | 115.6(5) |
| C(8)-N(3)-C(6) | 123.4(5) | C(8)-N(3)-C(5) | 121.2(5) |
| C(6)-N(3)-C(5) | 114.4(5) | C(8)-N(4)-N(5) | 112.4(4) |
| C(9)-N(5)-N(4) | 119.6(4) | C(9)-N(5)-Cu(1) | 116.6(4) |
| N(4)-N(5)-Cu(1) | 123.7(3) | C(14)-N(6)-C(10) | 119.6(5) |
| C(14)-N(6)-Cu(1) | 127.5(4) | C(10)-N(6)-Cu(1) | 112.8(4) |
| C(15)-N(7)-C(19) | 117.3(5) | C(15)-N(7)-Cu(1)#1 | 123.3(4) |
| C(19)-N(7)-Cu(1)#1 | 114.7(4) | O(2)-N(8)-O(3) | 122.5(5) |
| O(2)-N(8)-O(1) | 118.9(5) | O(3)-N(8)-O(1) | 118.6(5) |
| C(2)-C(1)-S(1) | 110.1(5) | C(1)-C(2)-N(1) | 117.3(6) |
| N(1)-C(3)-N(2) | 124.7(5) | N(1)-C(3)-S(1) | 115.3(4) |
| N(2)-C(3)-S(1) | 120.0(4) | C(5)-C(4)-N(2) | 115.6(6) |
| C(4)-C(5)-N(3) | 114.7(6) | C(7)-C(6)-N(3) | 115.1(7) |
| C(6)-C(7)-N(2) | 117.2(7) | N(4)-C(8)-N(3) | 115.7(5) |
| N(4)-C(8)-S(2) | 125.2(4) | N(3)-C(8)-S(2) | 119.2(4) |
| N(5)-C(9)-C(10) | 114.7(5) | N(5)-C(9)-C(15) | 123.1(5) |
| C(10)-C(9)-C(15) | 122.2(5) | N(6)-C(10)-C(11) | 121.1(5) |
| N(6)-C(10)-C(9) | 114.9(5) | C(11)-C(10)-C(9) | 124.0(5) |
| C(12)-C(11)-C(10) | 118.5(6) | C(13)-C(12)-C(11) | 119.6(6) |
| C(12)-C(13)-C(14) | 119.1(6) | N(6)-C(14)-C(13) | 122.0(6) |
| N(7)-C(15)-C(16) | 122.9(5) | N(7)-C(15)-C(9) | 116.3(5) |

Table S1 Continued

| | | | |
|---|---|---|---|
| C(16)-C(15)-C(9) | 120.9(5) | C(17)-C(16)-C(15) | 118.7(6) |
| C(16)-C(17)-C(18) | 119.3(6) | C(19)-C(18)-C(17) | 118.2(6) |

Symmetry transformations used to generate equivalent atoms: #1 $-x-2$, $-y+2$, $-z+1$

**Table S2**. The half-maximal inhibitory concentrations ($IC_{50}$) of H**L**, **1**, and **2** against different cell lines.

| | $IC_{50}$ (nmol/mL) | | |
|---|---|---|---|
| | H**L** | **1** | **2** |
| Hep-G2 | 78.83 | 38.11 | 16.86 |
| PLC/PRF/5 | 3.26 | 2.18 | $2.54\times10^{-5}$ |
| HuH-7 | 192.20 | 176.60 | 87.53 |

**Table S3**. The pharmacokinetic data for HL via i.v. administration with dosages of 0.5 mg $kg^{-1}$ in ICR mice (n = 3).

| Individual and mean plasma concentration-time data | | | | | | | | |
|---|---|---|---|---|---|---|---|---|
| Dose ($mg{\cdot}kg^{-1}$) | Dose route | Sampling time (h) | Concentration (ng/mL) | | | Mean (ng/mL) | SD | CV (%) |
| | | | 4 | 5 | 6 | | | |
| 0.50 | IV | 0.03 | 853.50 | 805.60 | 760.20 | 806.43 | 46.66 | 5.79 |
| | | 0.50 | 407.40 | 426.80 | 213.60 | 349.27 | 117.89 | 33.75 |
| | | 1.00 | 419.70 | 361.50 | 242.30 | 341.17 | 90.43 | 26.51 |
| | | 2.00 | 447.60 | 405.10 | 263.20 | 371.97 | 96.56 | 25.96 |
| | | 4.00 | 346.80 | 274.20 | 98.40 | 239.80 | 127.72 | 53.26 |
| | | 8.00 | 218.90 | 227.60 | 41.40 | 162.63 | 105.08 | 64.61 |
| | | 24.00 | 113.30 | 88.60 | 49.40 | 83.77 | 32.22 | 38.47 |
| PK Parameters | | Unit | 4 | 5 | 6 | Mean | SD | / |
| $R^2$ | | NA | 0.89 | 1.00 | 0.44 | 0.78 | 0.30 | 38.00 |
| $T_{1/2}$ | | h | 13.40 | 12.12 | 10.12 | 11.88 | 1.66 | 13.93 |
| $T_{max}$ | | h | 0.03 | 0.03 | 0.03 | 0.03 | 0.00 | 0.00 |
| $C_0$ | | ng/mL | 899.29 | 842.59 | 831.55 | 857.81 | 36.35 | 4.24 |
| $AUC_{last}$ | | h*ng/mL | 5547.17 | 5107.84 | 1987.97 | 4214.32 | 1940.5 | 46.05 |
| $AUC_{0\text{-}inf}$ | | h*ng/mL | 7738.05 | 6656.93 | 2709.11 | 5701.36 | 2647.1 | 46.43 |
| Vz_obs | | mL/kg | 1249.48 | 1313.23 | 2694.23 | 1752.31 | 816.35 | 46.59 |
| Cl_obs | | mL/h/kg | 64.62 | 75.11 | 184.56 | 108.10 | 66.43 | 61.45 |
| $MRT_{last}$ | | h | 8.15 | 7.85 | 7.55 | 7.85 | 0.30 | 3.81 |
| Vss_obs | | mL/kg | 1170.20 | 1177.58 | 2918.63 | 1755.47 | 1007.3 | 57.38 |

**Table S4**. The pharmacokinetic data for H**L** via PO administration with dosages of 30 mg kg$^{-1}$ in ICR mice (n = 3).

| Individual and mean plasma concentration-time data | | | | | | | | |
|---|---|---|---|---|---|---|---|---|
| Dose (mg·kg$^{-1}$) | Dose route | Sampling time (h) | Concentration (ng/mL) 1 | 2 | 3 | Mean (ng/mL) / | SD | CV (%) |
| 30 | PO | 0.50 | 280.8 | 350.5 | 95.9 | 242.40 | 131.6 | 54.3 |
| | | 1.00 | 400.3 | 264.1 | 613.4 | 425.93 | 176.1 | 41.3 |
| | | 2.00 | 452.9 | 314.0 | 462.6 | 409.83 | 83.1 | 20.3 |
| | | 4.00 | 408.7 | 221.3 | 317.2 | 315.73 | 93.7 | 29.7 |
| | | 8.00 | 253.0 | 163.0 | 236.7 | 217.57 | 48.0 | 22.0 |
| | | 24.00 | 226.5 | 114.6 | 93.3 | 144.80 | 71.6 | 49.4 |
| PK Parameters | | Unit | 1 | 2 | 3 | Mean | SD | / |
| $R^2$ | | NA | 0.21 | 0.83 | 1.00 | 0.68 | 0.4 | 61.1 |
| $T_{1/2}$ | | h | 30.06 | 23.26 | 11.49 | 21.61 | 9.4 | 43.5 |
| $T_{max}$ | | h | 2.00 | 0.50 | 1.00 | 1.17 | 0.8 | 65.5 |
| $C_{max}$ | | ng/mL | 452.90 | 350.50 | 613.40 | 472.27 | 132.5 | 28.1 |
| $AUC_{last}$ | | h*ng/mL | 6688.08 | 4055.03 | 5266.90 | 5336.67 | 1317.9 | 24.7 |
| $AUC_{0-inf}$ | | h*ng/mL | 16512.12 | 7901.48 | 6813.43 | 10409.01 | 5313.4 | 51.0 |
| Vz_F_obs | | mL/kg | 78802.61 | 127435.04 | 72984.82 | 93074.16 | 29899. | 32.1 |
| Cl_F_obs | | mL/h/kg | 1816.85 | 3796.76 | 4403.07 | 3338.89 | 1352.5 | 40.5 |
| $MRT_{last}$ | | h | 10.52 | 9.60 | 8.07 | 9.40 | 1.2 | 13.1 |

**Table S5**. The weight record of CD®(SD) IGS Rats as ministered orally with sterilizing solution (groups 1, 2), 5 mg kg⁻¹ (groups 3, 4), and 10 mg kg⁻¹ (groups 5, 6) of H**L**.

| | Group | Ear Tag | D1 | D2 | D3 | D4 | D5 | D6 | D7 | D8 | D9 | D10 | D11 | D12 | D13 |
|---|---|---|---|---|---|---|---|---|---|---|---|---|---|---|---|
| Sterilizing Solution | 1 | 1 | 182.5 | 178.9 | 189.5 | 195.1 | 196.4 | 190.2 | 194.3 | 208.9 | 209.5 | 216.2 | 218.8 | 230.1 | 234.1 |
| | | 2 | 193.2 | 191.2 | 196.9 | 191.0 | 200.9 | 205.7 | 207.6 | 211.4 | 210.7 | 216.5 | 222.8 | 224.0 | 225.8 |
| | | 3 | 189.4 | 193.3 | 204.1 | 208.3 | 215.2 | 218.6 | 225.1 | 232.6 | 235.2 | 242.3 | 244.7 | 250.3 | 260.9 |
| | AV | | 188.4 | 187.8 | 196.8 | 198.1 | 204.2 | 204.8 | 209.0 | 217.6 | 218.5 | 225.0 | 228.8 | 234.8 | 240.3 |
| | SD | | 5.4 | 7.8 | 7.3 | 9.0 | 9.8 | 14.2 | 15.4 | 13.0 | 14.5 | 15.0 | 13.9 | 13.8 | 18.3 |
| | 2 | 1 | 179.6 | 192.7 | 202.6 | 213.5 | 223.4 | 234.8 | 248.2 | 257.7 | 263.6 | 273.9 | 286.5 | 290.4 | 309.3 |
| | | 2 | 204.9 | 209.9 | 221.3 | 228.6 | 234.8 | 246.6 | 254.4 | 264.1 | 266.8 | 273.6 | 285.3 | 292.9 | 305.7 |
| | | 3 | 197.3 | 209.9 | 219.2 | 225.7 | 228.9 | 243.2 | 253.1 | 262.4 | 264.8 | 274.6 | 288.3 | 290.1 | 302.2 |
| | AV | | 193.9 | 204.2 | 214.4 | 222.6 | 229.0 | 241.5 | 251.9 | 261.4 | 265.1 | 274.0 | 286.7 | 291.1 | 305.7 |
| | SD | | 13.0 | 9.9 | 10.2 | 8.0 | 5.7 | 6.1 | 3.3 | 3.3 | 1.6 | 0.5 | 1.5 | 1.5 | 3.6 |
| HL 5 mg/kg (12.2 μmol/kg) | 3 | 4 | 189.1 | 186.5 | 198.3 | 199.2 | 204.6 | 204.5 | 211.1 | 209.3 | 199.7 | 183.6 | 178.8 | 179.1 | 181.3 |
| | | 5 | 191.2 | 193.0 | 193.1 | 203.8 | 205.1 | 215.4 | 214.8 | 210.2 | 216.9 | 227.8 | 229.7 | 226.4 | 227.2 |
| | | 6 | 187.7 | 185.7 | 193.9 | 197.8 | 196.9 | 194.1 | 204.6 | 207.8 | 211.0 | 203.5 | 212.3 | 216.5 | 219.3 |
| | AV | | 189.3 | 188.4 | 195.1 | 200.3 | 202.2 | 204.7 | 210.2 | 209.1 | 209.2 | 205.0 | 206.9 | 207.3 | 209.3 |
| | SD | | 1.8 | 4.0 | 2.8 | 3.1 | 4.6 | 10.7 | 5.2 | 1.2 | 8.7 | 22.1 | 25.9 | 24.9 | 24.5 |
| | 4 | 4 | 191.4 | 198.6 | 209.6 | 220.3 | 225.1 | 232.6 | 245.6 | 253.8 | 263.8 | 275.4 | 284.0 | 292.5 | 299.6 |
| | | 5 | 192.0 | 203.2 | 213.9 | 224.9 | 230.1 | 241.3 | 249.9 | 265.7 | 264.6 | 280.0 | 289.3 | 293.9 | 302.9 |
| | | 6 | 194.1 | 204.2 | 211.8 | 223.9 | 231.6 | 243.1 | 256.0 | 266.3 | 272.9 | 284.1 | 293.6 | 301.3 | 311.5 |
| | AV | | 192.5 | 202.0 | 211.8 | 223.0 | 228.9 | 239.0 | 250.5 | 261.9 | 267.1 | 279.8 | 289.0 | 295.9 | 304.7 |
| | SD | | 1.4 | 3.0 | 2.2 | 2.4 | 3.4 | 5.6 | 5.2 | 7.1 | 5.0 | 4.4 | 4.8 | 4.7 | 6.1 |
| HL 10 mg/kg (24.4 μmol/kg) | 5 | 7 | 191.0 | 185.1 | 198.5 | 202.4 | 204.7 | 206.9 | 200.8 | 194.1 | 197.2 | 190.1 | 193.6 | 191.3 | 197.5 |
| | | 8 | 190.4 | 187.1 | 202.5 | 207.8 | 199.9 | 189.7 | 186.4 | 168.8 | 157.8 | 140.2 | - | - | - |
| | | 9 | 180.9 | 177.2 | 190.5 | 190.4 | 186.3 | 187.8 | 172.1 | 156.4 | 145.9 | 134.5 | - | - | - |
| | AV | | 187.4 | 183.1 | 197.2 | 200.2 | 197.0 | 194.8 | 186.4 | 173.1 | 167.0 | 154.9 | 193.6 | 191.3 | 197.5 |
| | SD | | 5.7 | 5.2 | 6.1 | 8.9 | 9.5 | 10.5 | 14.4 | 19.2 | 26.9 | 30.6 | 111.8 | 110.4 | 114.0 |
| | 6 | 7 | 197.7 | 202.9 | 215.0 | 219.4 | 228.9 | 241.5 | 250.7 | 266.0 | 272.8 | 269.1 | 291.9 | 298.9 | 308.1 |
| | | 8 | 195.7 | 208.1 | 218.2 | 227.8 | 235.2 | 244.8 | 251.5 | 257.6 | 264.3 | 259.9 | 274.7 | 274.9 | 275.3 |
| | | 9 | 189.5 | 200.0 | 210.5 | 219.1 | 220.7 | 233.3 | 243.0 | 252.9 | 260.0 | 259.3 | 277.1 | 282.3 | 291.8 |
| | AV | | 194.3 | 203.7 | 214.6 | 222.1 | 228.3 | 239.9 | 248.4 | 258.8 | 265.7 | 262.8 | 281.2 | 285.4 | 291.7 |
| | SD | | 4.3 | 4.1 | 3.9 | 4.9 | 7.3 | 5.9 | 4.7 | 6.6 | 6.5 | 5.5 | 9.3 | 12.3 | 16.4 |

| | Group | Ear Tag | D14 | D15 | D16 | D17 | D18 | D19 | D20 | D21 | D22 | D23 | D24 | D25 | D26 | D27 |
|---|---|---|---|---|---|---|---|---|---|---|---|---|---|---|---|---|
| Sterilizing Solution | 1 | 1 | 234.4 | 240.4 | 243.6 | 241.5 | 235.9 | 235.1 | 231.5 | 225.6 | 230.6 | 231.2 | 236.6 | 234.6 | 235.1 | 239.3 |
| | | 2 | 230.7 | 232.3 | 235.7 | 236.7 | 240.3 | 242.0 | 246.8 | 244.2 | 241.2 | 242.9 | 247.5 | 254.7 | 251.6 | 247.3 |
| | | 3 | 261.5 | 263.6 | 265.6 | 269.8 | 270.5 | 265.1 | 274.6 | 279.1 | 284.0 | 279.2 | 288.6 | 293.5 | 299.9 | 303.9 |
| | AV | | 242.2 | 245.4 | 248.3 | 249.3 | 248.9 | 247.4 | 251.0 | 249.6 | 251.9 | 251.1 | 257.6 | 260.9 | 262.2 | 263.5 |
| | SD | | 16.8 | 16.2 | 15.5 | 17.9 | 18.8 | 15.7 | 21.9 | 27.2 | 28.3 | 25.0 | 27.4 | 29.9 | 33.7 | 35.2 |
| | 2 | 1 | 318.8 | 320.5 | 326.0 | 340.7 | 347.7 | 349.1 | 353.9 | 364.4 | 366.5 | 373.4 | 383.1 | 393.8 | 401.0 | 402.3 |
| | | 2 | 314.7 | 314.1 | 328.9 | 334.2 | 343.3 | 350.2 | 358.1 | 366.5 | 379.2 | 375.9 | 386.5 | 395.6 | 401.2 | 407.3 |
| | | 3 | 306.9 | 316.5 | 324.9 | 330.7 | 335.8 | 341.7 | 352.2 | 357.3 | 367.0 | 373.5 | 389.5 | 392.3 | 402.6 | 397.7 |
| | AV | | 313.5 | 317.0 | 326.6 | 335.2 | 342.3 | 347.0 | 354.7 | 362.7 | 370.9 | 374.3 | 386.4 | 393.9 | 401.6 | 402.4 |
| | SD | | 6.0 | 3.2 | 2.1 | 5.1 | 6.0 | 4.6 | 3.0 | 4.8 | 7.2 | 1.4 | 3.2 | 1.7 | 0.9 | 4.8 |
| HL 5 mg/kg (12.2 μmol/kg) | 3 | 4 | 177.3 | 177.0 | - | - | - | - | - | - | - | - | - | - | - | - |
| | | 5 | 233.1 | 233.7 | 241.9 | 238.4 | 226.5 | 220.9 | 213.6 | 204.5 | 205.8 | 192.8 | 197.6 | 198.3 | 198.6 | 207.3 |
| | | 6 | 213.4 | 222.1 | 224.9 | 227.6 | 221.6 | 232.9 | 234.1 | 235.0 | 234.1 | 230.1 | 242.3 | 238.0 | 243.8 | 237.3 |
| | AV | | 207.9 | 210.9 | 233.4 | 233.0 | 224.1 | 226.9 | 223.9 | 219.8 | 220.0 | 211.5 | 220.0 | 218.2 | 221.2 | 222.3 |
| | SD | | 28.3 | 30.0 | 135.0 | 134.6 | 129.4 | 131.1 | 129.6 | 127.8 | 127.8 | 123.5 | 128.9 | 127.5 | 129.7 | 129.2 |
| | 4 | 4 | 305.1 | 316.9 | 321.7 | 333.5 | 339.6 | 347.6 | 352.0 | 361.5 | 365.9 | 372.6 | 386.3 | 386.8 | 399.0 | 404.0 |
| | | 5 | 309.8 | 320.1 | - | - | - | - | - | - | - | - | - | - | - | - |
| | | 6 | 320.6 | 329.6 | 335.4 | 348.8 | 356.8 | 367.9 | 373.5 | 382.0 | 387.8 | 393.6 | 400.9 | 406.9 | 409.9 | 412.1 |
| | AV | | 311.8 | 322.2 | 328.6 | 341.2 | 348.2 | 357.8 | 362.8 | 371.8 | 376.9 | 383.1 | 393.6 | 396.9 | 404.5 | 408.1 |
| | SD | | 7.9 | 6.6 | 189.8 | 197.1 | 201.2 | 206.8 | 209.7 | 214.9 | 217.8 | 221.4 | 227.4 | 229.3 | 233.6 | 235.6 |
| HL 10 mg/kg (24.4 μmol/kg) | 5 | 7 | 202.5 | 203.5 | - | - | - | - | - | - | - | - | - | - | - | - |
| | | 8 | - | - | - | - | - | - | - | - | - | - | - | - | - | - |
| | | 9 | - | - | - | - | - | - | - | - | - | - | - | - | - | - |
| | AV | | 202.5 | 203.5 | - | - | - | - | - | - | - | - | - | - | - | - |
| | SD | | 116.9 | 117.5 | 0.0 | 0.0 | 0.0 | 0.0 | 0.0 | 0.0 | 0.0 | 0.0 | 0.0 | 0.0 | 0.0 | 0.0 |
| | 6 | 7 | 314.9 | 324.3 | 330.3 | 345.5 | 351.4 | 358.9 | 364.2 | 374.1 | 372.9 | 377.8 | 378.5 | 387.9 | 368.4 | 364.5 |
| | | 8 | 254.4 | 231.4 | 199.4 | - | - | - | - | - | - | - | - | - | - | - |
| | | 9 | 299.6 | 305.6 | 315.2 | 325.1 | 330.1 | 334.3 | 339.4 | 343.4 | 349.1 | 346.3 | 345.1 | 344.0 | 350.4 | 358.9 |
| | AV | | 289.6 | 287.1 | 281.6 | 335.3 | 340.8 | 346.6 | 351.8 | 358.8 | 361.0 | 362.1 | 361.8 | 366.0 | 359.4 | 361.7 |
| | SD | | 31.5 | 49.1 | 71.6 | 193.9 | 197.0 | 200.5 | 203.5 | 207.7 | 208.8 | 209.6 | 209.6 | 212.4 | 207.7 | 208.8 |
